# Stimulating TAM-mediated anti-tumor immunity with mannose-decorated nanoparticles in ovarian cancer

**DOI:** 10.1186/s12885-022-09612-2

**Published:** 2022-05-06

**Authors:** Evan B. Glass, Alyssa A. Hoover, Kennady K. Bullock, Matthew Z. Madden, Bradley I. Reinfeld, Whitney Harris, Dominique Parker, Demetra H. Hufnagel, Marta A. Crispens, Dineo Khabele, W. Kimryn Rathmell, Jeffrey C. Rathmell, Andrew J. Wilson, Todd D. Giorgio, Fiona E. Yull

**Affiliations:** 1 Department of Biomedical Engineering, Vanderbilt University, Nashville, TN USA; 2 Department of Pharmacology, Vanderbilt University, Nashville, TN USA; 3 Department of Pathology, Microbiology and Immunology, Vanderbilt University Medical Center, Nashville, TN USA; 4 Vanderbilt University School of Medicine, Nashville, TN USA; 5 Department of Medicine, Vanderbilt University Medical Center, Nashville, TN USA; 6 Program in Cancer Biology, Vanderbilt University, Nashville, TN USA; 7 Department of Obstetrics and Gynecology, Vanderbilt University Medical Center, Nashville, TN USA; 8 Vanderbilt Center for Immunobiology and Vanderbilt-Ingram Cancer Center, Vanderbilt University Medical Center, Nashville, TN USA; 9 Department of Obstetrics and Gynecology, Washington University School of Medicine, St. Louis, MO USA; 10 Department of Chemical and Biomolecular Engineering, Vanderbilt University, Nashville, TN USA

## Abstract

**Background:**

Current cancer immunotherapies have made tremendous impacts but generally lack high response rates, especially in ovarian cancer. New therapies are needed to provide increased benefits. One understudied approach is to target the large population of immunosuppressive tumor-associated macrophages (TAMs). Using inducible transgenic mice, we recently reported that upregulating nuclear factor-kappaB (NF-κB) signaling in TAMs promotes the M1, anti-tumor phenotype and limits ovarian cancer progression. We also developed a mannose-decorated polymeric nanoparticle system (MnNPs) to preferentially deliver siRNA payloads to M2, pro-tumor macrophages in vitro. In this study, we tested a translational strategy to repolarize ovarian TAMs via MnNPs loaded with siRNA targeting the inhibitor of NF-κB alpha (IκBα) using mouse models of ovarian cancer.

**Methods:**

We evaluated treatment with MnNPs loaded with IκBα siRNA (IκBα-MnNPs) or scrambled siRNA in syngeneic ovarian cancer models. ID8 tumors in C57Bl/6 mice were used to evaluate consecutive-day treatment of late-stage disease while TBR5 tumors in FVB mice were used to evaluate repetitive treatments in a faster-developing disease model. MnNPs were evaluated for biodistribution and therapeutic efficacy in both models.

**Results:**

Stimulation of NF-κB activity and repolarization to an M1 phenotype via IκBα-MnNP treatment was confirmed using cultured luciferase-reporter macrophages. Delivery of MnNPs with fluorescent payloads (Cy5-MnNPs) to macrophages in the solid tumors and ascites was confirmed in both tumor models. A three consecutive-day treatment of IκBα-MnNPs in the ID8 model validated a shift towards M1 macrophage polarization in vivo. A clear therapeutic effect was observed with biweekly treatments over 2-3 weeks in the TBR5 model where significantly reduced tumor burden was accompanied by changes in immune cell composition, indicative of reduced immunosuppressive tumor microenvironment. No evidence of toxicity associated with MnNP treatment was observed in either model.

**Conclusions:**

In mouse models of ovarian cancer, MnNPs were preferentially associated with macrophages in ascites fluid and solid tumors. Evidence of macrophage repolarization, increased inflammatory cues, and reduced tumor burden in IκBα-MnNP-treated mice indicate beneficial outcomes in models of established disease. We have provided evidence of a targeted, TAM-directed approach to increase anti-tumor immunity in ovarian cancer with strong translational potential for future clinical studies.

**Supplementary Information:**

The online version contains supplementary material available at 10.1186/s12885-022-09612-2.

## Background

The use of cancer immunotherapies, including immune checkpoint blockades (ICBs), is rapidly expanding as a result of early successes in clinical trials. Multiple clinical trials have resulted in FDA-approved treatments for a variety of cancers, including melanoma [1, 2], non-small cell lung cancer [3], urothelial cancer [4, 5], renal cell carcinoma [6], colorectal cancer [7], and triple-negative breast cancer (TNBC) [8], among others reviewed here [9]. While ICBs represent an important advancement in cancer therapy, disappointingly low overall response rates (ORRs) are commonly observed. In particular, clinical trials involving the treatment of epithelial ovarian cancer resulted in ORRs of 15 and 8% for monotherapies of nivolumab and pembrolizumab, respectively [10–12]. Epithelial ovarian cancer (EOC) is estimated to be the fifth leading cause of cancer-related deaths in females in the U.S. and is in desperate need of improved therapies [13]. The relatively late presentation of disease in most patients contributes to overall survival of 40% at diagnosis [14]. The primary standard of care (surgery followed by platinum- and taxane-based chemotherapy) is initially effective in over 70% of patients, but only half of these patients exhibit non-detectable levels of cancer cells 5 months after treatment [15]. Even in those patients, small numbers of chemotherapy-resistant cells can remain dormant in the peritoneal cavity for several months before growing exponentially [15]. However, as with many other types of cancer, ovarian cancer is a prime candidate for macrophage-specific immunotherapy.

Tumor-associated macrophages (TAMs) are the most abundant immune cell in most types of cancer, including epithelial ovarian cancer [16, 17]. TAMs are prevalent in both the solid tumor as well as the ascites fluid associated with ovarian cancer progression. Ascites development is correlated with severity of ovarian cancer (present in over one-third of patients) and is linked to poor disease prognosis as well as development of chemoresistance [18–20]. TAMs in both the tumor and ascites promote an immunosuppressive tumor microenvironment (TME), hinder the efficacy of ICB, and drive tumor progression and metastasis [21, 22]. Several techniques to alter the TME, including TAM ablation or limiting TAM recruitment, have been attempted with moderate successes [23, 24]. However, a potentially more robust method leverages macrophage plasticity. This phenomenon provides the opportunity to target M2-like immunosuppressive TAMs and “repolarize” them into an M1-like pro-inflammatory, anti-tumor phenotype. Repolarizing TAMs for cancer immunotherapy is a rapidly growing field of study with encouraging early results [25, 26].

Our lab has previously identified the nuclear factor-κB pathway (NF-κB) as a target for repolarizing macrophages both in vitro and in vivo since this pathway drives macrophage phenotype [27, 28]. This work provides evidence that modulating macrophage phenotype in mouse cancer models induces beneficial therapeutic outcomes. Using transgenic mice, we have recently demonstrated M1 macrophage repolarization, increased cytotoxic T cell responses, and reduced tumor burden in syngeneic mouse models of ovarian cancer [28]. The goal of the present study is to evaluate a new translational approach involving a targeted nanoparticle-based delivery of a siRNA cargo to specifically stimulate NF-κB activity in ovarian TAMs.

Our group has extensive experience designing nanoparticle formulations for delivering siRNA to cells in vivo [29–31]. We have previously used a small interfering RNA (siRNA) sequence specific for the inhibitor of NF-κB alpha (IκBα). Because IκBα functions to inhibit the canonical NF-κB pathway, our previous work confirmed that delivering siRNA against IκBα can increase activity of the canonical pathway and induce M1 polarization [27, 29].

Although siRNA-based therapies are promising, delivery platforms are crucial to their success because oligonucleotides are unable to cross cell membranes and are rapidly cleared from circulation when administered as unformulated macromolecules [32]. Targeted formulations can enhance delivery of functional siRNA to the appropriate cells while simultaneously minimizing activity in non-target cells and tissues [33, 34]. By combining a siRNA against IκBα with a targeted delivery system, we aim to increase the specificity of M1 activation to the tumor microenvironment and limit off-target effects elsewhere. The phenotypic diversity of macrophages, often characterized by the differing expression levels of various surface receptors, allows for targeting via the CD206 macrophage mannose receptor, which is overexpressed on M2-polarized macrophages and TAMs [35]. The overexpression of CD206 on immunosuppressive TAMs allows for targeting with mannose-decorated delivery systems [25, 27, 36]. Specifically, polymers designed to form nanoscale micelles containing siRNA and decorated with mannose enable the targeted delivery of RNA interference (RNAi) therapies that modulates macrophage phenotype [27]. These micelles are spherical with diameters of 100-150 nm to promote clathrin-mediated endocytosis into the targeted macrophages, which has been extensively documented [37–39]. Our group has previously demonstrated MnNP targeting to macrophages using a mannosylated triblock copolymer [36, 40]. This system has since been improved via the development of a diblock copolymer that is more reproducible and simpler to fabricate [41]. The mannose-decorated diblock copolymer was shown to successfully target M2-polarized bone marrow-derived macrophages (BMDMs) and repolarize them to an M1 phenotype [27].

Mannosylated nanoparticle (MnNP) delivery systems can be especially beneficial in the context of ovarian cancer. EOCs with high levels of immunosuppressive TAMs, in both the solid tumor and ascites, result in poor prognosis for patients while cases with elevated M1-polarized macrophages are correlated with increased overall survival and progression-free survival [42, 43]. Furthermore, intraperitoneal (IP) injection of nanoparticles allows for localized delivery with limited risk for off-target accumulation or rapid clearance that is often associated with intravascular (IV) delivery [30]. The potential for targeted repolarization of TAMs in EOC led to the hypothesis that IP injection of MnNPs loaded with IκBα siRNA (IκBα-MnNP) will locally target and reprogram macrophages to induce anti-tumor immunity in mouse models of ovarian cancer.

This study follows-up on our previous work by utilizing the optimized MnNPs to deliver IκBα siRNA to TAMs in mouse models of ovarian cancer. Two models of ovarian cancer were used to evaluate the effects of IκBα-MnNPs on TAMs. The ID8 ovarian tumors on a C57Bl/6 background enabled examination of treating late-stage disease while the TBR5 ovarian tumors on an FVB background allowed for evaluation of early-stage treatment in an aggressive disease model. MnNP biodistribution was evaluated to confirm limited off-target delivery in both models. Tumor-bearing mice were treated with IκBα siRNA-loaded MnNPs, scrambled siRNA-loaded MnNPs, or PBS to evaluate changes in tumor burden in terms of tumor weight and ascites accumulation. The safety profile of the two treatment regimens was also evaluated to characterize any toxicities caused by MnNP administration. Immune cell composition, macrophage phenotypes, and cytokine signaling were evaluated to investigate the mechanisms leading to differential outcomes and, ultimately, to demonstrate the effects of TAM repolarization on tumor suppression.

## Materials and methods

### Materials

All materials were purchased from Sigma-Aldrich unless otherwise noted. Monomethyl ether hydroquinone inhibitors were removed from dimethylaminoethyl methacrylate (DMAEMA) and butyl methacrylate (BMA) using an activated basic aluminum oxide column. All DNA and RNA oligonucleotides were purchased from Integrated DNA Technologies (Coralville, IA). For in vivo biodistribution studies, double stranded DNA (dsDNA) designed to be length-matched to therapeutic IκBα siRNA and conjugated with a cyanine-5 (Cy5) fluorophore was used. IκBα siRNA sequence was based on previous studies and the scrambled siRNA sequence was randomized from the IκBα sequence and analyzed via the Basic Local Alignment Search Tool (BLAST, NCBI) to ensure no off-target effects with our sequence [27, 29]. Oligonucleotide sequences are listed in Supplemental Table 1.

### Polymer synthesis

The mannose-poly (ethylene glycol)-(DMAEMA-co-BMA) (MnPEGDB) polymer was fabricated as previously described [27, 36, 40, 41]. Nucleophilic substitution of propargyl bromide with D-mannose to form mannose-alkyne was performed and characterized previously [27]. The core of the micelle comprises a diblock copolymer fabricated using 4-cyano-4-(ethylsulfanylthiocarbonyl)-sulfanylpentanoic acid (ECT) as a chain transfer agent (CTA) conjugated to an azide-functionalized PEG (Az-PEG). The Az-PEG-ECT was then RAFT polymerized to DMAEMA and BMA as previously described to create a “smart” polymer capable of encapsulating anionic siRNA and inducing endosomal escape upon uptake [27, 41, 44]. The AzPEGDB was then reacted with Mn-alkyne (1:3 azide:alkyne molar ratio) via copper-catalyzed azide-alkyne cycloaddition (CuAAC) using a previously optimized copper catalyst concentration of 0.75 mM with 5 mM of sodium ascorbate [27]. Chemical structures and ^1^H-NMR for all reaction steps are shown in Supplemental Figs. S1-4, and mannose conjugation was verified with Fourier transform infrared (FTIR) spectroscopy as previously described (Supplemental Fig. S5) [27]. All chemical structures were made using ChemDraw (PerkinElmer). FTIR spectroscopy was performed at the Vanderbilt Institute of Nanoscale Science and Engineering (VINSE) and all ^1^H-NMR experiments were performed at the Vanderbilt Small Molecule NMR Facility on a 400 MHz spectrometer (Bruker).

### Nanoparticle preparation

Nanoscale polymeric complexes were fabricated as previously described [27, 30, 41]. To form mannosylated-nanoparticles (MnNPs) with oligonucleotides, MnPEGDB was dissolved in 90% (v/v) 10 mM citrate buffer (pH = 4) with 200-proof ethanol (EtOH). MnNPs were complexed with either Cy5-dsDNA, IκBα siRNA, or scrambled siRNA for 30 minutes to form micelles before adding 5× volume 10 mM phosphate buffer (pH = 8) for a final solution pH of 7.4. The micelle N^+^/P^−^ ratio, determined by mole ratio of protonated amines on the DMAEMA polymer to the number of phosphates on the oligonucleotides, was chosen as 10:1 based on previous studies [27, 41]. Nanoparticle size and zeta potential were evaluated using a Malvern Zetasizer located at VINSE, and the results are shown in Supplemental Fig. S6. All in vitro treatments were conducted with a final concentration of 50 nM oligonucleotides and all in vivo treatments were performed using a dose of 1 mg/kg (1 mg of oligonucleotide/kg of mouse weight). For in vivo NP preparation, the pH = 7.4 solution was diluted in sterile phosphate buffered saline (PBS) without magnesium and calcium and centrifuged in 5000 MWCO Amicon® Ultra-15 Centrifugal Filters (Millipore Sigma; UFC905024) at 2000×g for 30 minutes. The concentrated NPs were diluted in PBS (−/−) and centrifuged again before adding PBS to get to the appropriate volume for a 1 mg/kg concentration. The final preparation was filtered through a 0.45 μm syringe filter before being used for intraperitoneal injection.

### Cell culture and tumor induction

Unless otherwise noted, all cells were cultured in Dulbecco’s Modified Eagle’s Medium (DMEM, low glucose, pyruvate, Gibco; 11,885,084) supplemented with 10% fetal bovine serum (FBS, certified, Gibco; 16,000,044) and 1% penicillin/streptomycin (P/S) at 37 °C in a 5% CO_2_ humidified atmosphere. Luciferized ID8 ovarian tumor cells and TBR5 genetically modified ovarian tumor cells were used as previously described [45–48]. TBR5 cells were from Dr. Sandra Orsulic [49] and luciferized ID8 cells were from Dr. Balkwill [50]. All animal work was reviewed and approved by the Vanderbilt University Institutional Animal Care and Use Committee (IACUC). The ID8 cells were used in syngeneic C57Bl/6 background mice while TBR5 cells were used in syngeneic FVB background mice. For tumor induction, cells were resuspended in sterile PBS at a concentration of 5 × 10^6^ cells/200 μL. A sterile 3 mL Luer-Lok™ syringe with an 18G needle was used to inject of 5 × 10^6^ cells tumor cells in 200 μL PBS intraperitoneally (IP) into each mouse. At endpoint, all mice were euthanized via carbon dioxide inhalation with secondary cervical dislocation as approved by IACUC protocols.

Primary bone marrow-derived macrophages (BMDMs) were isolated from healthy female wild-type FVB mice and immortalized NGL-BMDMs were previously derived from NF-κB green fluorescent protein (GFP)-luciferase (NGL) reporter transgenic mice on an FVB background [51–53]. BMDMs were used for background in vitro assays and M2-polarized BMDMs are commonly used as surrogates for TAMs as a practical substitute [27, 29, 54].

### Culturing and treating bone marrow-derived macrophages

Immortalized NGL-BMDMs were used from frozen stocks for in vitro experiments [53]. NGL-BMDMs were plated in 6-well plates at 1 × 10^6^ cells/well in 2 mL of DMEM (ThermoFisher; 11,995,073) supplemented with 10% FBS and 1% P/S. The macrophages were polarized to M1 with 10 ng/mL each of IFN-γ and LPS for 24 hours and to M2 with 10 ng/mL of IL-4 for 48 hours. M2-polarized NGL-BMDMs were treated with Scr-MnNPs or IκBα-MnNPs for 24 hours before collecting cells for luminescent measurements. Samples were prepared for luminescence readings using a Luciferase Assay System (Promega; E4030) following the manufacturer’s instructions.

### In vivo biodistribution studies in ovarian tumor-bearing mice

For the preliminary 24-hour delivery study, 6 female C57Bl/6 mice were injected IP with 5 × 10^6^ ID8 ovarian tumor cells in 200 μL PBS. Tumors developed for 8 weeks before treatment. Control mice were injected with 200 μL PBS and treatment mice received 200 μL PBS containing MnNPs loaded with Cy5-dsDNA at the previously listed dosage. After 24 hours, the mice were sacrificed and the ascites, tumors, and spleens were collected. The ascites was centrifuged at 1500 rpm for 5 minutes, supernatant collected, and red blood cells (RBCs) lysed with 5 mL Geyz lysing buffer (4.15 g NH_4_Cl, 0.5 g KHCO_3_ in 500 mL MilliQ water) for 5 minutes at 37 °C. This step was repeated as needed until a clear pellet was obtained. The resulting cells were resuspended in PBS with 1% BSA and snap-frozen in liquid nitrogen for RNA isolation or protein analysis. The solid tumors were collected into 3 mL DMEM (MT-10-13-CV) containing 10% FBS and 1% P/S for 1 hour on ice. The tissue was cut into small pieces and resuspended in 3 mL DMEM with 200 μL Collagenase A (Roche; 10,103,578,001), 300 μL hyaluronidase (Sigma; H4272), 500 μL DNase I (Sigma; D5025), and 30 μL amphotericin B (ThermoFisher; 15,290,026) and placed at 37 °C for 2 hours with frequent vortexing. The solution was filtered through a 70 μm strainer to form a single cell suspension used for flow cytometry. Tumors were then treated with the same RBC lysis buffer as the ascites. Similarly, spleens were collected in DMEM (10% FBS, 1% P/S) for 1 hour on ice, chopped into small pieces, and immediately filtered through a 70 μm strainer twice. The final single cell suspension was treated with RBC lysis buffer before flow analysis.

The long-term biodistribution study was performed in the TBR5 ovarian tumor model. 10 female FVB mice received IP injections of 5 × 10^6^ TBR5 cells in 200 μL PBS (day 0). Tumors developed for 7 days before starting treatment on day 7. Mice either received IP injections of 100 μL PBS or 100 μL Cy5-MnNPs. Treatments were performed on days 7, 10, 14, and 17 before takedown on day 18. Single cell suspensions were made from tumors, ascites, and spleen as previously described and used for flow cytometry analysis.

### Flow Cytometry of in vivo biodistribution

Single cell suspensions were obtained from the tumors, ascites fluid, and spleens of either ID8 or TBR5 tumor-bearing mice. For ID8 24-hour biodistribution, the cells were resuspended in flow buffer (PBS with 2 mM EDTA and 0.5% (v/v) BSA) at 1 × 10^6^ cells/50 μL buffer. The following anti-mouse antibodies were used: CD45 PE-Cy7 (eBioscience; 25-0451-82), F4/80 PE (eBioscience; 12-4801-82), and Gr-1 Alexa Fluor 700 (eBioscience; 53-5931-82). After staining for 30 minutes, the cells were rinsed in PBS and resuspended in flow buffer before running flow analysis at the Vanderbilt Flow Cytometry Shared Resource. All flow analysis was performed using FlowJo v10.7.1. Flow gating strategy is shown in Supplemental Fig. S7.

For the TBR5 biodistribution study, flow cytometry was performed as previously described [55]. The cells were incubated in an Fc block (BD Biosciences; 553,142) for 10 minutes at RT, stained for surface markers for 15 minutes at RT, washed with a FACS buffer containing PBS with 2% (v/v) FBS, and resuspended in the FACS buffer for flow analysis on a Miltenyi MACSQuant Analyzer 10 or 16. The eBioscience™ Foxp3/transcription factor staining buffer kit (Fisher Scientific; 00-5523-00) was used for intracellular staining. After surface staining, the cells were fixed and permeabilized for 20 minutes at 4 °C before staining for intracellular markers for 30 minutes at 4 °C. To quantify cell viability, a Ghost Dye Red 780 viability marker (1:4000, Cell Signaling Technology; 18452S) was used. The following anti-mouse antibodies were used: CD45 BV510 (1:1600, BioLegend; 103,138), CD3 FITC (1:200, BioLegend; 100,204), CD4 PerCP-Cy5.5 (1:600, BioLegend; 100,540), CD8a PE (1:800, eBioscience; 12-0081-82), B220 e450 (1:400, ThermoFisher; 48-0452-82), NKp46 PE-Cy7 (1:200, BioLegend; 137,618), CD11c PE (1:1000, BioLegend; 117,308), CD11b e450 (1:1600, ThermoFisher; 48-0112-82), F4/80 PE-Cy7 (1:800, BioLegend; 123,114), Ly6C FITC (1:4000, BioLegend; 128,006), and Ly6G PerCP-Cy5.5 (1:800, BioLegend 127,616). Flow cytometry data were analyzed with FlowJo v10.7.1. Representative gating strategies of ascites, tumors, and spleens are shown in Supplemental Figs. S8-10.

### In vivo tumor studies

Treatment of the ID8 ovarian tumor model was formulated as a late-stage disease treatment. Similar to uptake studies, 5 × 10^6^ ID8 ovarian tumor cells in 200 μL PBS were IP injected into 15 female C57Bl/6 mice (day 0) and allowed to develop tumors for 7 weeks. Starting on day 49, mice received IP injections of 100 μL PBS, Scr-MnNPs, or IκBα-MnNPs for 3 consecutive days. MnNPs were given at a dose of 1 mg/kg. The mice were euthanized 1 day after the final treatment. Blood samples were collected for liver (aspartate aminotransferase, AST, and alanine aminotransferase, ALT) and kidney (blood urea nitrogen, BUN) enzyme measurements and the ascites volume was measured and collected. Normal ranges for serum AST, ALT, and BUN levels were referenced from the Vanderbilt University Translational Pathology Shared Resource (TPSR). Tumors and spleens were then harvested. The ascites fluid was collected, centrifuged, and the supernatant stored for protein serum concentration analysis as described above. The remaining ascites cell pellet was further processed with RBC lysis as described above until the cell pellet was clear. The cell pellet was frozen at − 80 °C for RNA isolation. Tumors were weighed, cut in half, and one half was snap frozen in liquid nitrogen for RNA isolation. The other tumor half and the entire spleens were fixed in 10% formalin for 48-72 hours for histology before being switched to 70% EtOH. Processing, embedding and sectioning, and hematoxylin and eosin (H&E) staining of tumor tissue, as well as blood chemistry analyses, were performed by the Vanderbilt University Medical Center (VUMC) Translational Pathology Shared Resource (TPSR) core. H&E-stained tissues were imaged using the EVOS XL Core microscope (ThermoFisher) on 4x and 10x brightfield magnification.

The faster developing TBR5 model was used to model a more aggressive, early-stage treatment strategy. 5 × 10^6^ TBR5 ovarian tumor cells in 200 μL PBS were IP injected into 15 female FVB mice (day 0) and allowed to develop tumors for 7 days. A biweekly treatment was adopted to combat the aggressive growth. Mice received IP injections of 100 μL PBS, Scr-MnNPs, or IκBα-MnNPs on days 7, 10, 14, 17, and 21 and the mice were sacrificed on day 22. For the extended study, additional MnNP treatments were administered on days 24 and 28 before euthanizing mice on day 29. Similar to the ID8 model, blood samples and ascites were collected immediately after takedown before surgically removing the spleens and tumors. The ascites was centrifuged and supernatant collected. The tumors were weighed and then split into two samples: one for fixation and one for snap freezing for RNA isolation. Spleens were fixed as previously described. The same analyses performed on the ID8 ovarian tumors were repeated here. For flow cytometry analysis, CD3 APC (1:200, BioLegend; 100,236) and CD8a AF488 (1:1600, BioLegend; 100,723) were used in place of CD3 FITC and CD8a PE used for biodistribution. The rest of the panel was the same with the addition of the following anti-mouse antibodies: CD206 APC (1:500, BioLegend; 141,708), FOXP3 PE (1:125, ThermoFisher; 12-5773-82), and PD-1 PE (1:100, BioLegend; 135,206). Flow gating strategy was repeated as previously shown. To visualize tumor cells populations, cells were gated on forward scatter (FSC) vs side scatter (SSC), single cells (FSC-area vs FSC-height), live/dead, and CD45-. The CD45- cells were visualized again as FSC vs SSC where a clear population of “big” SSC-high (SSChi) cells were present only in cells from the solid tumors and ascites, but not the spleen. Representative tumor cells gating is shown in Supplemental Fig. S11.

### Immunofluorescent staining for confocal imaging of tumor sections

The protocol of immunofluorescent staining and analysis has been previously described [28]. To evaluate CD8 T cell infiltration, the primary antibody rat anti-mouse CD8 (Novus Biologicals; BP1-49045SS, 1:100) was used with a secondary goat anti-rat IgG Alexa Fluor 488 (Abcam; ab150157). Previously sectioned tumor samples were deparaffinized in Xylenes 2x for 10 minutes each. The samples were then rehydrated in 100% EtOH 2x for 2 minutes each, and then once each in 90, 80, and 70% EtOH (v/v in DI water) for 2 minutes. The slides were then incubated on a shaker in 0.1% Sudan Black Dye solution (diluted in 70% EtOH) for 20 minutes before one more 2-minute wash in 50% EtOH. The sections were then permeabilized in 1X Tris-buffered saline (TBS) with 0.1% Tween 20 (TBST) for 20 minutes, rinsed in TBS for 5 minutes, and finally rinsed briefly with Milli-Q DI water. Antigen retrieval was performed by placing slides in a rice cooker with a 10 mM sodium citrate solution (pH = 6.0) with 0.1% Tween 20 and heated for 15-20 minutes before cooling at RT for 30 minutes. The slides were then washed 3x with TBS for 5 minutes each. Slides were treated with a blocking buffer comprised of 4 mL 0.5% TBST, 0.04 g bovine serum albumin, and 250 μL goat serum (Abcam; ab7481) for 1 hour in a humidified chamber at RT. The blocking buffer was aspirated and 100 μL of primary antibody was added and incubated at 4 °C overnight. The primary antibody was aspirated and each slide washed 3x for 10 minutes each in TBST and then rinsed a final time with TBS for 5 minutes while shaking. The TBS was aspirated and 100 μL of secondary antibody was diluted in blocking buffer (1:1000) and incubated at RT in the dark for 2 hours. The slides were washed 3x for 10 minutes each in TBST followed by a single 5-minute wash in TBS on a shaker. DAPI (0.1 μ/mL) was added to each slide and incubated at RT for 5 minutes to stain cell nuclei. The slides were washed 2x for 3 minutes each in TBS and then 20 μL ProLong™ Gold Antifade Mountant (ThermoFisher; P36930) was added to preserve fluorescent signal. Slides were stored at 4 °C until imaging was performed via fluorescent microscopy (Nikon C1si + confocal microscope system on Nikon Eclipse Ti-0E inverted microscope base, Plan APO VC 20× objective, 405/488 dichroic mirror). Images were analyzed using Fiji in ImageJ [56].

### RNA extraction for quantitative reverse transcription polymerase chain reaction (qRT-PCR)

For in vitro BMDM experiments, RNA was isolated using the RNeasy Mini Kit (Qiagen; 74,106) and residual DNA was removed using the RNase-Free DNase set (Qiagen; 79,256). cDNA was synthesized with SuperScript IV reverse transcriptase kit (Invitrogen; 18,090,050). Quantitative reverse transcription polymerase chain reaction (qRT-PCR) was performed using SsoAdvanced Universal SYBR Green Supermix (Bio-Rad; 1,725,270) on a CFX96 real-time PCR instrument and software (Bio-Rad) through the VUMC Molecular Cell Biology Resource (MCBR) core.

For in vivo experiments, RNA was extracted from both the ascites cells and tumor cells using TRIzol™ (Invitrogen; 15,596,026) and a Direct-zol RNA Miniprep kit (Zymo Research; R2050). Snap frozen tumor samples were ground into small pieces with a mortar and pestle before suspending in 300 μL TRIzol solution. The ascites pellet was resuspended in 300 μL TRIzol solution. Both solutions were used with the Direct-zol RNA Miniprep kit following the manufacturer’s instructions. Final RNA concentration was measured with a NanoDrop 200 spectrophotometer (Biotek). cDNA fabrication and qRT-PCR were performed as described above. All primer sequences are listed in Supplemental Table 2. For all experiments, target genes were normalized to a housekeeping gene (B2M or GAPDH) to obtain the ΔCT value. All qRT-PCR data is shown as relative expression using the 2^-ΔΔCT^ method.

### Western blot

Protein isolated from ascites cells in the TBR5 experimental model was used for western blot analysis of IκBα expression. Whole protein isolation, western blotting, and signal detection were performed as previously described [57]. Primary antibodies used were rabbit polyclonal anti-IκBα (1:100 dilution, Cell Signaling Technology; 9242). Equal loading was confirmed using mouse monoclonal anti-histone H3 (1:1000 dilution, Cell Signaling Technology; 14269S) as a loading control. To image loading control and experimental antibody on the same blot, the gel was cut prior to hybridization. The pieces of the uncropped blot are shown in Supplemental Fig. S12.

### Statistical analysis

All statistical analyses were performed using a one-way ANOVA with Tukey’s multiple comparison test in the case of two or more groups, a two-way ANOVA with Tukey’s multiple comparison test in the case of two or more groups in two or more sets, or a two-tailed student’s t-test in the case of only two groups, all with α = 0.05. Statistical analyses were performed using GraphPad Prism v8.4.3. All figures were made using Adobe Photoshop 2020 v21.2.2.

## Results

### Quantifying changes in NF-κB activation in MnNP-treated macrophages

**Fig. 1:**
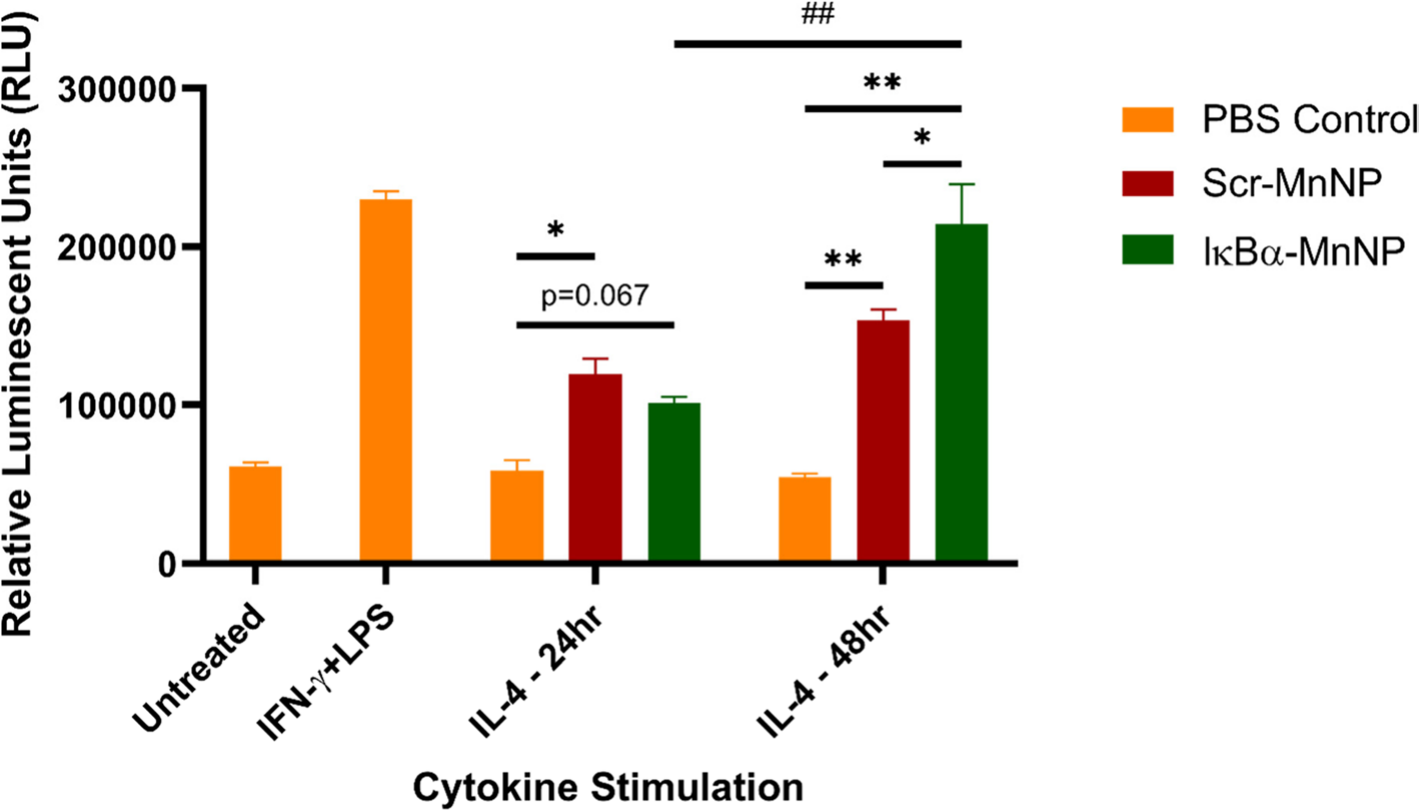
NF-κB Activation in MnNP-Treated Polarized NGL-BMDMs. BMDMs isolated from NGL reporter mice were polarized to M1 (IFN-γ + LPS) or M2 (IL-4, 24 or 48 hours) or left unpolarized (M0) and examined for luminescent readout. The M2-polarized BMDMs were also treated with MnNPs for 24 hours before measuring luminescence. For the 24 hour-polarized M2 BMDMs, the Scr- and IκBα-MnNP treatments both increased NF-κB activation, but this effect was more pronounced in the 48-hour IL-4-treated BMDMs, especially with the IκBα-MnNP (*n* = 3, *p < 0.05 **p < 0.001 ##p < 0.001)

Knockdown of IκBα by targeted siRNA results in activation of the canonical NF-κB pathway known to promote inflammation through the M1 polarization of macrophages [58, 59]. To assess activation of canonical NF-κB following treatment with IκBα siRNA loaded into MnNPs, immortalized BMDMs derived from NF-κB green fluorescent protein (GFP)-luciferase (NGL) reporter transgenic mice (FVB background) were cultured [29, 51]. The NGL-BMDMs express a GFP-luciferase fusion protein in response to NF-κB activation allowing for luminescence as a method to evaluate effects of treatment in the context of differently polarized macrophages. BMDMs were polarized to an M2 phenotype via interleukin-4 (IL-4) stimulation for 24 or 48 hours before treating with MnNPs for 24 hours. Control groups of M1-polarized or unpolarized (M0) BMDMs were also included. M1 polarization was induced via treatment with interferon-γ (IFN-γ) and lipopolysaccharide (LPS) for 24 hours. For the 48 hour-stimulated M2 BMDMs, the IκBα-MnNP treatment significantly increased luminescence compared to both the PBS control and the Scr-MnNP treatment, indicating activation of canonical NF-κB (Fig. 1). M2 macrophages treated with MnNPs loaded with IκBα siRNA activated NF-κB to levels equivalent to M1 macrophages treated with PBS as estimated by luminescence intensity, indicating a robust shift in macrophage phenotype induced by IκBα-MnNP treatment. Interestingly, for the 24 hour-stimulated M2 BMDMs, both Scr-MnNP and IκBα-MnNP treatments increased luminescence, indicating potential activation of canonical NF-κB due to the mannosylated carriers themselves. There was no significant change in luminescent intensity for Scr-MnNP treatments between 24- and 48-hour IL-4 stimulation, while the IκBα-MnNP treatment significantly increased luminescent intensity for the longer stimulated M2 macrophages.

### Biodistribution analysis of MnNPs in a late-stage ID8 ovarian tumor model

**Fig. 2:**
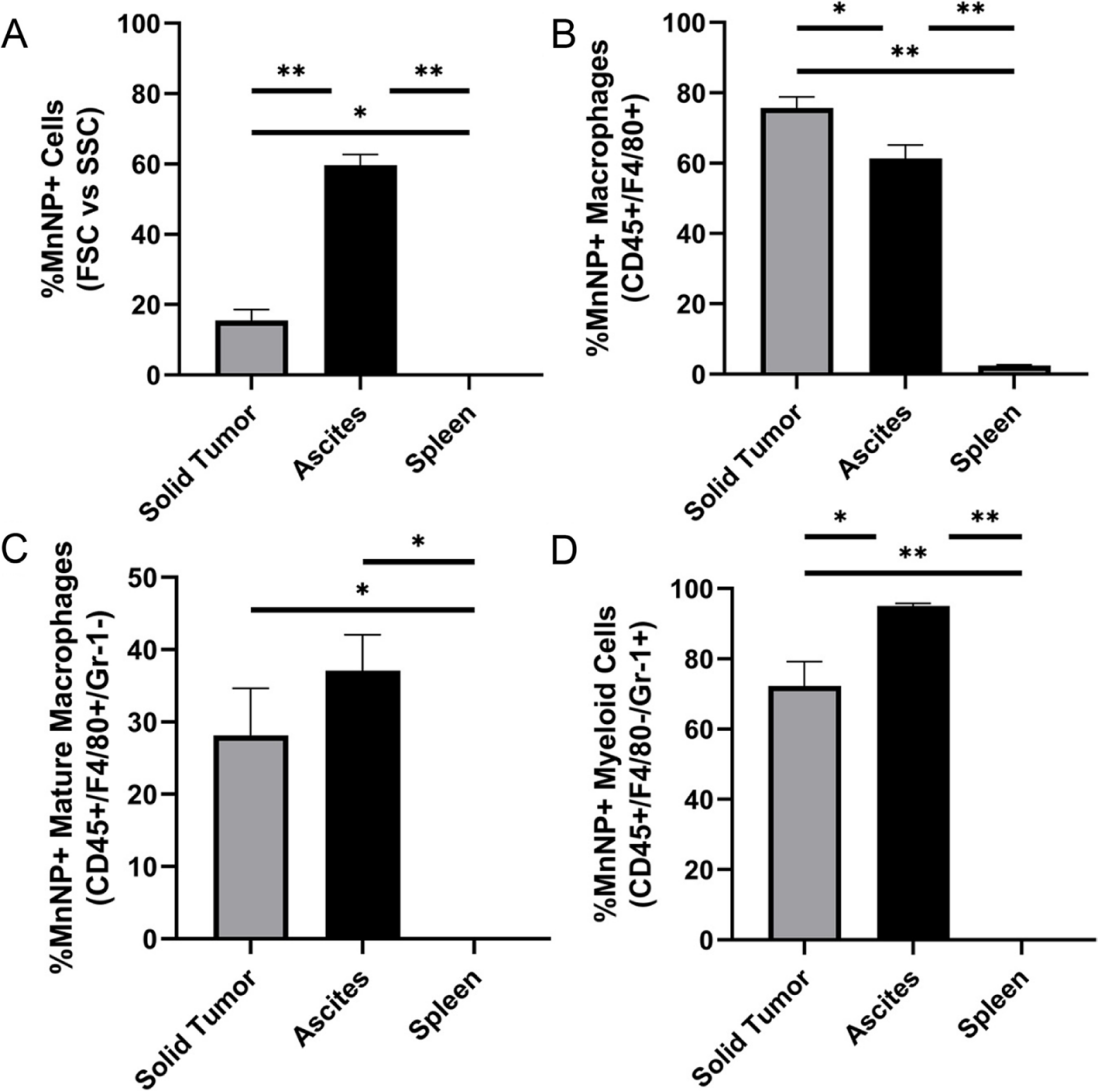
MnNP Biodistribution in ID8 Tumor Model. C57Bl/6 mice bearing ID8 ovarian tumors were treated IP with Cy5-MnNPs. After 24 hours, the ascites, tumors, and spleens were collected for flow cytometry analysis. **A** The general cell population gated on FSC vs. SSC revealed a high percentage of MnNP+ cells in the ascites and almost 20% of cells in the tumor, but negligible delivery to any cells in the spleen. **B** Gating on CD45+/F4/80+ macrophages revealed that about 60% of macrophages in the ascites and ~ 75% of TAMs were MnNP+. Additional gating for (**C**) Gr-1- mature macrophages and (**D**) Gr-1+ myeloid cells also revealed high percentages of MnNP uptake in the ascites and tumor (*n* = 3, *p < 0.05 **p < 0.001)

Biodistribution studies were conducted to evaluate in vivo delivery of MnNPs to specific cell populations. Female C57Bl/6 mice received intraperitoneal (IP) injections of 5 × 10^6^ ID8 ovarian tumor cells and were monitored for tumor development for 8 weeks. The ID8 model is slow-developing (> 7 weeks) and is often used for studying late-stage ovarian cancer [47, 60, 61]. These mice received a single IP injection of MnNPs loaded with Cy5-labeled double-stranded DNA (Cy5-MnNPs) 8 weeks after tumor induction. The ascites fluid, tumors, and spleens were collected from the mice 24 hours after MnNP administration and processed for flow cytometry. Cy5 fluorescence was used to estimate MnNP biodistribution. The general cell population was gated on forward-scatter (FSC) vs. side-scatter (SSC). Immune cells were gated on CD45 followed by F4/80 vs. Gr-1 to visualize immune cell subsets. The MnNPs were located almost exclusively in the tumor and ascites with 15.4% of cells in the tumor and 59.7% of cells in the ascites gating positive for Cy5 compared to < 1% of cells in the spleens (Fig. 2A). Additional gating on the general macrophage population (CD45+/F4/80+) revealed that 75.7% of TAMs and 61.3% of the macrophages in the ascites were associated with Cy5-MnNPs, consistent with the anticipated delivery of cargo to these cell types in the peritoneal cavity (Fig. 2B). Gr-1 gating demonstrated the same trends in both mature macrophage (F4/80+/Gr-1-) and myeloid cell (F4/80−/Gr-1+) populations, with high percent uptake in the tumor and ascites and negligible delivery to the spleen (Fig. 2C, D). These results suggest that even a single IP injection of MnNPs examined after 24 hours is able to deliver the payload to high percentages of macrophages in the tumors and ascites.

### Endpoint MnNP treatment of late-stage disease reveals encouraging effects

**Fig. 3:**
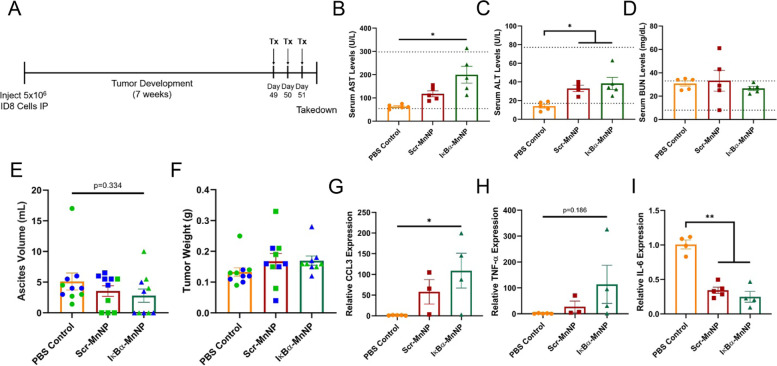
Endpoint Analysis of ID8 Tumors Treated with Therapeutic MnNPs. **A** Treatment schematic for development of ID8 ovarian tumor model and 3-day MnNP treatment. Serum levels of (**B**) AST, (**C**) ALT, and (**D**) BUN were evaluated at endpoint. AST and ALT were significantly elevated in IκBα-MnNP treatment, but still within normal ranges while BUN levels were unchanged (*n* = 5, *p < 0.05). Changes in (**E**) ascites volume and (**F**) tumor weight at takedown (*n* = 10, color indicates experimental groupings). RNA isolated from the ascites cells revealed increases in the inflammatory cytokines (**G**) CCL3 and (**H**) TNF-α (PBS control *n* = 5, Scr-MnNP *n* = 3, IκBα-MnNP *n* = 4, *p < 0.05). **I** Cells in the ascites also significantly decreased in expression of IL-6 in both MnNP treatments (PBS control and IκBα-MnNP *n* = 4, Scr-MnNP *n* = 5, *p < 0.05)

To evaluate the anti-tumor efficacy of MnNP treatments in late-stage disease, ID8 ovarian tumor cells were injected into the peritoneal cavity of female C57Bl/6 mice and allowed to progress for 7 weeks. Once these mice approached humane endpoint, MnNP treatments were administered by IP injection daily for 3 consecutive days. The treatment groups included a PBS control or MnNPs containing the Scr siRNA (Scr-MnNPs) or the IκBα siRNA (IκBα-MnNPs). Mice were euthanized 24 hours after the final injection (Fig. 3A). This model was chosen because ID8 ovarian tumors are known to produce high ascites volumes, which comprise large populations of immunosuppressive macrophages, and reflect a significant subpopulation of human ovarian cancers [48]. In this way, the ID8 model could be used to evaluate the effects of MnNP-mediated siRNA delivery to immunosuppressive macrophage populations in hopes of seeing changes in the immune cells. Additionally, a 3-consecutive day treatment at endpoint was used to evaluate toxicity of multi-day MnNP injections. Importantly, limited toxicity was observed in mice treated with MnNPs with serum levels of aspartate aminotransferase (AST), alanine aminotransferase (ALT), and blood urea nitrogen (BUN) all falling within normal ranges (indicated as horizontal dotted lines) despite slight elevation in AST levels in the IκBα-MnNP treatment (Fig. 3B-D). While the 3-day MnNP treatment did not result in toxicity, there was also no significant change in tumor burden as evaluated by ascites volume and tumor weight at endpoint (Fig. 3E, F). The trend of decreasing ascites volume from 5.1 ± 1.4 mL in the control to 2.8 ± 1.1 mL in the IκBα-MnNP treatment provided encouragement that beneficial changes were occurring, but this effect was minimal due to the late-stage treatment of advanced disease. However, hematoxylin and eosin (H&E) staining of tumor sections revealed visible differences in the treatment groups, most noticeably areas of decreased epithelial cellularity that could result from an increase in cell death (Supplemental Fig. S13). For cells collected from the ascites, there was a significant increase in RNA expression of the M1 marker C-C motif chemokine ligand 3 (CCL3) and a trend of increased tumor necrosis factor-α (TNF-α) expression in the IκBα-MnNP treatment compared to control (Fig. 3G, H). Finally, there was a significant decrease in immunosuppressive interleukin-6 (IL-6) expression in both MnNP treatments (Fig. 3I). Expression of an additional M1 marker C-X-C motif ligand 9 (CXCL9), a chemokine responsible for attracting CD8 T cells, trended upward in the ascites, while CCL3 and TNF-α expression in cells from solid tumors also trended upward but not significantly (Supplemental Fig. S14).

### Biweekly treatment of ovarian tumors reveals macrophage-specific delivery in ascites and solid tumors

**Fig. 4:**
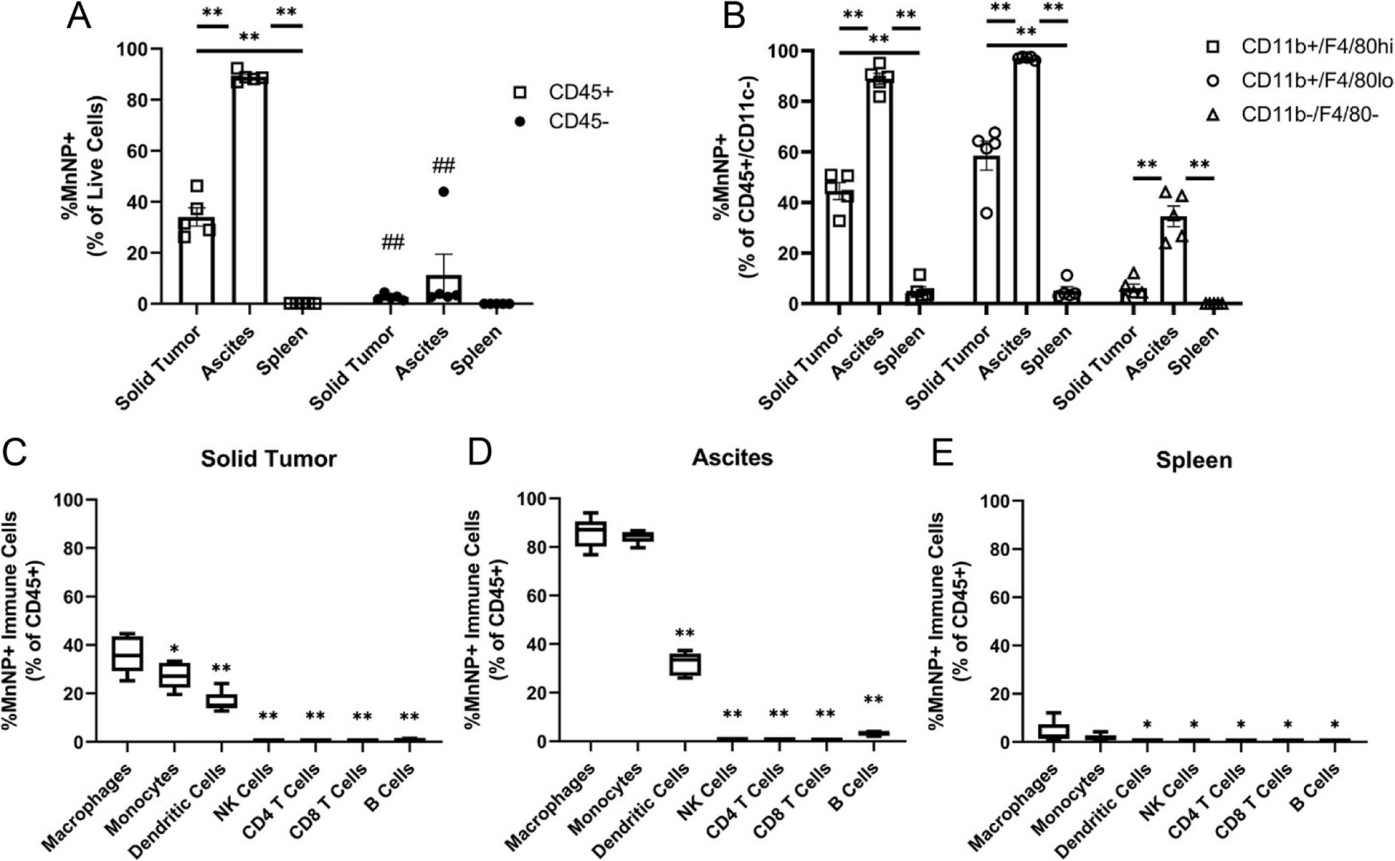
Biodistribution of Biweekly MnNP Treatment in TBR5 Model. MnNP biodistribution in the TBR5 ovarian tumor model was examined via flow cytometry. **A** Cy5-MnNPs demonstrated significant increase in uptake in CD45+ immune cells in both the ascites and solid tumor compared to the CD45- populations (*n* = 5, ##*p* < 0.001). MnNPs were also associated with over 89% of CD45+ cells in the ascites and 30% of CD45+ cells in the solid tumor (**p < 0.001). **B** Direct comparison of the different organs for macrophages, monocytes, and other immune cells revealed targeted delivery to the tumors and ascites with minimal off-target delivery to the spleens (*n* = 5, **p < 0.001). Macrophages displayed significantly elevated %MnNP+ levels compared to most other immune cells in the (**C**) tumor, (**D**) ascites, and (**E**) spleen. The %MnNP+ macrophage population was significantly higher than all other immune cell subtypes in the tumor, and higher than all other subtypes except monocytes in the ascites and spleen (*n* = 5, *p < 0.05, **p < 0.001)

To evaluate the effects of repetitive IκBα-MnNP treatment on a more aggressive tumor model, the TBR5 ovarian model in FVB mice was adopted. Biodistribution was assessed to confirm preferential in vivo delivery of MnNPs to macrophage populations in the ascites and solid tumor. The TBR5 model exhibits rapid disease progression (over the course of 28 days) and provides a platform to characterize the impact of a feasible treatment schedule in an aggressive model. Due to rapid progression, a biweekly (twice per week) treatment regimen for 2 weeks (total of 4 treatments) was used. Cy5-MnNPs were delivered via IP injection to evaluate biodistribution. One day after the final treatment, the ascites, solid tumors, and spleens were collected and processed for flow cytometry analysis. Initial gating on the immune cell population (CD45) revealed that 89% of CD45+ immune cells in the ascites and 34.1% of CD45+ immune cells in the solid tumor were positive for Cy5-MnNPs compared to 11.4 and 2.6% uptake in the non-immune cell (CD45-) populations of the ascites and tumors, respectively (Fig. 4A). Additionally, there was negligible uptake (< 1%) in any cells in the spleen, supporting our hypothesis that IP delivery can abrogate the off-target delivery normally associated with IV treatment.

Additional gating on specific immune cell populations was performed to examine macrophage targeting. Lymphocyte gating included CD45+/CD3+/CD4+ and CD45+/CD3+/CD8a + markers for CD4 and CD8 T cells, respectively. B cells were gated CD45+/CD3−/B220+ and natural killer (NK) cells were gated CD45+/CD3−/NKp46+. Myeloid populations were gated CD45+/CD11c+/CD11b- for dendritic cells (DCs) and CD45+/CD11c−/CD11b + for macrophages (F4/80hi) and monocytes (F4/80lo). Macrophages and monocytes were further gated Ly6C+/Ly6G- to remove neutrophils and eosinophils [62]. When examining delivery to the macrophage (CD11b+/F4/80hi), monocyte (CD11b+/F4/80lo), and other immune cell (CD11b−/F4/80-) populations in the different organs, the Cy5-MnNPs were almost exclusively localized to the macrophages and monocytes in the ascites and tumors with negligible uptake in the spleen (Fig. 4B). In tumors, MnNP delivery was significantly greater in macrophages compared to all other immune cell populations (Fig. 4C). Additionally, 85.7% of macrophages and 84.3% of monocytes in the ascites were Cy5-MnNP+, which was significantly elevated compared to all other immune cell populations (Fig. 4D). Finally, only 3.9% of macrophages in the spleen exhibited uptake of Cy5-MnNPs, demonstrating limited off-target delivery (Fig. 4E). These results demonstrate the intended targeting to TAMs as well as macrophages in the ascites with minimal off-target delivery to other immune cells or organs. Additionally, the biweekly treatment adopted for the aggressive tumor model exhibited greater MnNP uptake than observed in the previously used late-stage ID8 model.

### Delivery of IκBα-MnNPs to TAMs prevents ascites accumulation and alters immune cell phenotype

**Fig. 5:**
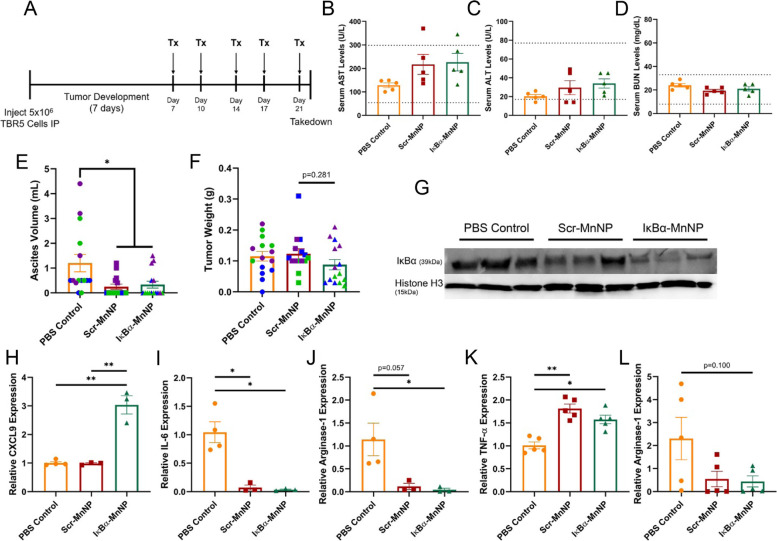
Endpoint Analysis of Biweekly MnNP Treatment in TBR5 Model. **A** Treatment schematic for development of TBR5 ovarian tumor model and biweekly MnNP treatment. Serum was collected at endpoint to evaluate circulating levels of (**B**) AST, (**C**) ALT, and (**D**) BUN (*n* = 5). Changes in (**E**) ascites volume and (**F**) tumor weight at endpoint (*n* = 15, *p < 0.05). **G** Western blot analysis of cells in the ascites revealed changes in IκBα protein levels (cropped image of western bands). RNA was collected from ascites cells and examined for expression of (**H**) CXCL9, (**I**) IL-6, and (**J**) Arginase-1 (PBS Control *n* = 4, Scr- and IκBα-MnNP *n* = 3, *p < 0.05, **p < 0.001). RNA collected from tumor cells was examined for expression of (**K**) TNF-α and (**L**) Arginase-1 (*n* = 5, *p < 0.05, **p < 0.001)

The TBR5 ovarian tumor model was used to elucidate the effects of IκBα-MnNP treatment on an aggressive ovarian tumor model. After tumor cell injection on day 0, MnNPs were injected on days 7, 10, 14, 17, and 21 before mice were humanely sacrificed on day 22 (Fig. 5A). Serum was collected and evaluated for AST, ALT, and BUN levels as previously described and, similar to the ID8 model, there was no detectable toxicity as a result of MnNP treatment (Fig. 5B-D). In contrast to the ID8 model, MnNP treatment in the TBR5 model significantly decreases ascites accumulation and slightly decreases tumor burden (Fig. 5E, F). Additionally, the weight gain associated with ascites fluid buildup in the PBS control group was abrogated in the MnNP-treated mice (Supplemental Fig. S15).

Similar to the ID8 model, there were clear indications of changes in tumor tissue as evaluated by H&E staining of tumor sections. The PBS control tumor appeared to only contain healthy cells while the Scr-MnNP treatment revealed some indications of depleted tumor cell populations based on observations in decreased cellularity (Supplemental Fig. S16). Meanwhile, the IκBα-MnNP treatment appeared to result in immune cell infiltration as evidenced by the increase in darker stained areas in the tumor sections (Supplemental Fig. S16). In both the ascites and tumor cells, there was a trend of decreasing IκBα RNA expression in mice treated with IκBα-MnNPs, but this change was not significant (Supplemental Fig. S17). Worthy of note was the observation of a relative increase in IκBα expression between the PBS control and the Scr-MnNP treatment in the ascites, which was abrogated by IκBα-MnNP treatment. However, there was a clear decrease in IκBα protein levels in the cells isolated from the ascites, indicating functionality of the IκBα siRNA in vivo (Fig. 5G). Delivery of IκBα-MnNPs also significantly increased expression of CXCL9 (M1 marker) and decreased Arginase-1 expression (M2 marker), while both MnNP treatments significantly decreased IL-6 expression (Fig. 5H-J). Importantly, MnNP treatment in TBR5 mice also significantly increased expression of the inflammatory cytokine TNF-α in the tumor, indicating a shift in the tumor microenvironment (Fig. 5K). Finally, there was a 5.2-fold decrease in Arginase-1 expression in the IκBα-MnNP treatment compared to control (Fig. 5L). Immunofluorescent (IF) staining of tumor sections for CD8 T cells was used to visualize T cell infiltration into treated tumors, which suggested an increase in CD8 T cells in tumors treated with IκBα-MnNPs (Supplemental Fig. S18).

### Extended IκBα-MnNP treatment alters immune cell populations and provides robust anti-tumor immune response

**Fig. 6:**
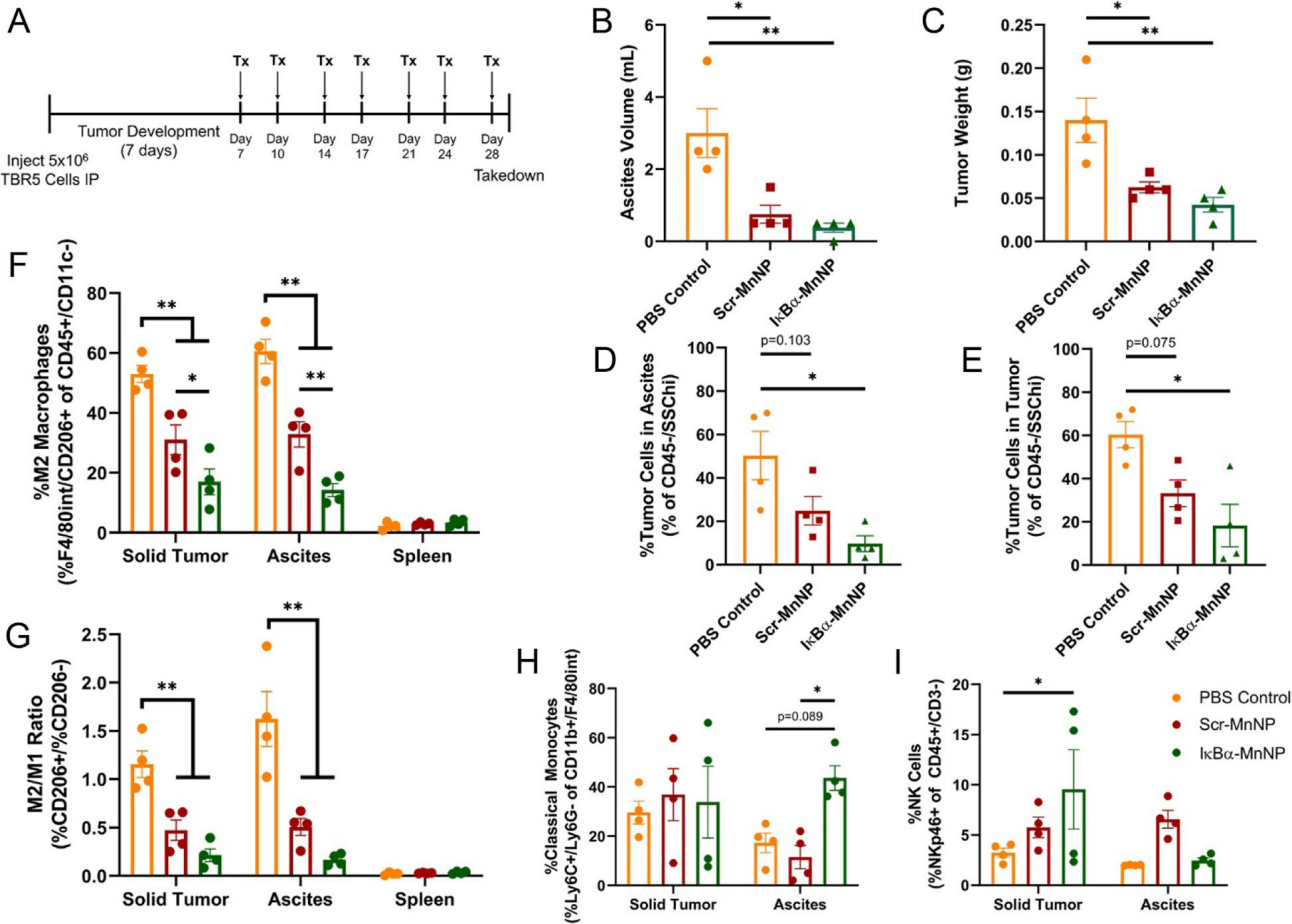
Increased Therapeutic Benefit in Extended MnNP Treatment Model. **A** Treatment schematic for extended MnNP treatment in the TBR5 tumor model. Changes in (**B**) ascites volume and (**C**) tumor weight at endpoint (*n* = 4, *p < 0.05, **p < 0.001). Flow cytometry gating on CD45−/SSChi cells revealed significant changes in the percent of tumor cells in the (**D**) ascites and **(E**) tumors. Cells gated on F4/80int/CD206+ revealed significant decreases in (**F**) the percent of M2-like TAMs and (**G**) the ratio of M2 (CD206+):M1 (CD206-) TAMs. The cells also revealed significant increases in (**H**) classical monocytes (F4/80int/Ly6C+/Ly6G-) in the ascites and (**I**) NK cells (CD3−/B220−/NKp46+) in the tumor (*n* = 4, *p < 0.05, **p < 0.001)

An extended MnNP treatment in the TBR5 model was used to further evaluate treatment effects on tumor progression and immune cell composition. TBR5 cells were injected IP and treatments started on day 7 and administered twice per week for 7 total treatments (Fig. 6A). The extended treatment significantly reduced both ascites volume accumulation and final tumor weight in both MnNP treatments, with a slightly more pronounced effect in the IκBα-MnNP treatment (Fig. 6B, C). The weight gain associated with ascites development was again abrogated similar to the previous study (Supplemental Fig. S19). Flow cytometry analysis was conducted to evaluate changes in immune cell populations after treatment. The percent of tumor cells (gated CD45−/SSChi) in the ascites and tumor was significantly decreased in the IκBα-MnNP treatment compared to the PBS control (Fig. 6D, E). Similar to previous results, the Scr-MnNP treatment led to a non-significant decrease in the percent of tumor cells compared to the control, indicating a slight therapeutic effect of the MnNPs themselves. Furthermore, the immune cells in the tumor and ascites were altered due to MnNP treatments. The percent of M2-like TAMs (F4/80int/CD206+) was significantly decreased by IκBα-MnNP treatment in both the tumor and ascites compared to both the PBS control and Scr-MnNP treatment (Fig. 6F). The Scr-MnNP treatment significantly decreased the percent of M2-like TAMs compared to the control, but not to the level of IκBα-MnNPs. Similarly, the ratio of M2/M1-like TAMs was significantly decreased in both MnNP treatments, with the effect being more pronounced in the IκBα-MnNP treatment (Fig. 6G). Additional analyses revealed that IκBα-MnNP treatment significantly increased the percent of classical (M1-like) monocytes (F4/80int/Ly6C+/Ly6G-) in the ascites and significantly increased the percent of NK cells (CD3−/B220−/NKp46+) in the tumor (Fig. 6H, I). Quantification of all immune cell populations also revealed trends of increasing CD8 T cells and dendritic cells while the percent of CD4 T cells was decreased (Supplemental Fig. S20). Taken together these results demonstrate the robust anti-tumor effects of IκBα-MnNP treatment due to altering the tumor immune microenvironment.

## Discussion

Repolarizing TAMs for cancer immunotherapy is a rapidly growing field of study with encouraging early results [25, 26]. Our group has previously identified RNA interference (RNAi) as an ideal candidate for altering TAM phenotype [29]. We have also demonstrated the anti-tumor effects of repolarizing TAMs by activating canonical NF-κB specifically in macrophages [28]. One of the primary challenges associated with translating this successful approach to intact living systems is the need for strongly preferential delivery of the siRNA payload to TAMs in vivo. Our group has made tremendous progress in developing a polymeric nanoparticle system capable of targeting M2-like TAMs while simplifying and optimizing the fabrication process [27, 36, 40]. Additionally, our combined expertise in studying ovarian tumors along with the potential benefits of relatively localized delivery via IP injections led to our decision to evaluate MnNP treatment in models of ovarian cancer [48]. Additionally, Zhang, et al., recently demonstrated ovarian tumor regression following IP administration of a different polymeric nanoparticle system loaded with mRNA encoding interferon regulatory factor 5 (IRF-5) to TAMs. However, this delivery system produced significant off-target IRF-5 activation in the spleens, causing M1 macrophage activation outside the TME [25]. In this study, we demonstrate the advantages of treating ovarian tumor-bearing mice with siRNA-loaded MnNPs to evaluate in vivo targeting to TAMs, changes in immune cell composition, and subsequent effects on tumor progression.

The initial in vitro study was designed to evaluate the ability of IκBα siRNA to activate the canonical NF-κB pathway in M2-polarized BMDMs. We had previously shown the knockdown of IκBα RNA expression and protein levels in M2 BMDMs treated with IκBα-MnNPs [27]. This study demonstrated that the knockdown of IκBα in BMDMs also corresponded with increased canonical NF-κB activation as evaluated by luminescent readout. This study was also the first to reveal the potential for the mannosylated carrier to stimulate pro-inflammatory effects in macrophages even with an inert siRNA payload. While the IκBα-MnNP increased luminescent readout to the level of the M1 control BMDMs, the Scr-MnNP also had an intermediate effect in increasing NF-κB activation. This result is consistent with other reports of inert particles decorated with mannose modulating macrophage phenotype and promoting inflammation with modest anti-tumor effects [25, 63]. Jaynes, et al., have recently shown that even specific binding to the CD206 mannose receptor alters the receptor conformation and leads to subsequent changes in macrophage phenotype [64]. This phenomenon can provide extra benefit for our purposes as we consistently observed a synergistic effect of the MnNP loaded with IκBα siRNA producing a robust repolarization of macrophages to a pro-inflammatory phenotype.

After establishing NF-κB activation in vitro, the MnNPs were used to treat two separate models of ovarian cancer. These models were chosen to evaluate two forms of treatment: late-stage treatment of advanced disease (ID8) and early-stage, repetitive treatment of an aggressive disease model (TBR5). To examine biodistribution in the late-stage model, the mice developed tumors until close to humane endpoint (based on swelling due to ascites buildup) and then a single MnNP dose was administered 24 hours before collection. These results revealed that even 24 hours after a single treatment, MnNPs preferably associated with macrophages in the ascites and tumor with no targeting to the spleen. This experiment confirmed our hypothesis that an IP injection for treating ovarian tumors could nullify the concerns often associated with IV injection of nanoparticles. Furthermore, the addition of the mannose moiety on the NPs prevented any off-target delivery to the spleen observed by other groups using decorated NP systems [25]. By injecting directly into the peritoneal cavity, the MnNPs were immediately exposed to the desired cell population in the target organ where they deliver a payload with improved specificity mediated via active endocytosis due to mannose conjugation. One of the major drawbacks with IV delivery of nanoparticles is the reliance on the enhanced permeability and retention (EPR) effect. While the EPR effect has been a cornerstone of developing nanomaterials therapies to target solid tumors, recent evidence shows the many challenges still hinder tumor targeting [65–67]. Furthermore, to ensure a therapeutic dose actually reaches the tumor, higher concentrations of nanoparticles are needed for IV delivery compared to IP, which is already localized to the tumor site. These biodistribution results validate our strategy for targeting TAMs in ovarian tumors via IP injections, and they demonstrate the reduced off-target delivery due to mannose conjugation which promotes active macrophage uptake in the ascites and tumor.

To evaluate therapeutic efficacy of MnNP treatment in ID8 tumors, we administered treatments on three consecutive days. One of the primary concerns with multi-day treatments was the toxicity potentially associated with nanoparticle injection. However, all treatments resulted in serum measurements within normal ranges indicating the safety of consecutive day treatments. This result is important as this regimen could be necessary for treating late-stage disease with a minimal timeframe available for treatment. These results from treating the late-stage model with therapeutic IκBα-MnNPs were encouraging because, although tumor burden was not significantly affected because of the late-stage treatment, the trend of decreased ascites volume provides positive signs of a therapeutic effect. The potential for reducing ascites accumulation after only 3 days of treatment at late stages of disease progression is promising for the future of TAM-targeted immunotherapies, especially as a potential treatment for overcoming chemoresistance. Ascites development is commonly associated with ovarian cancer (present in over one-third of patients), is linked to worse disease prognosis, and contributes to the development of chemoresistance due to the large population of immunosuppressive cells [18–20]. These early indications reveal treatment with MnNPs can alter the tumor microenvironment in ways that may provide synergy with other approved therapies to produce more pronounced anti-tumor effects. Also, although tumor weight did not change, positive anti-tumor effects were observed in H&E staining of the solid tumor indicating the MnNPs could reach the tumor and cause some histological changes, even following only 3 days of treatment.

Furthermore, positive effects were seen in the changes of RNA expression in ascites cells, such as the significant increase in M1 marker CCL3 and the significant decrease in IL-6. The decrease in IL-6 expression is important since this cytokine is released by immunosuppressive TAMs and has a direct stimulatory effect on ovarian tumor cells [68, 69]. Additionally, the observed increase in IκBα expression in the Scr-MnNP treatment compared to control indicates the effect of the negative feedback loop of IκBα production [59]. Consistent with our other findings, the Scr-MnNP treatment leading to an increased M1 response would subsequently cause an increase in IκBα production as the feedback loop in the macrophages is activated. The decrease in IκBα expression between the Scr-MnNP and IκBα-MnNP groups reveals the potential mechanism leading to a synergistic repolarization of TAMs. While both treatments potentially cause M1-induced inflammation, the IκBα-MnNP treatment prevents the feedback loop of producing more IκBα from occurring, allowing for a more robust macrophage repolarization. There were also trends of increasing CCL3 and TNF-α expression in the tumor cells, indicating a slight pro-inflammatory effect in the solid tumors. These results suggest that the IκBα-MnNPs are altering the TME in the ascites, but at this timepoint in response to a short-term treatment, the solid tumor is not significantly altered. These results align with our observations of ascites volume decreasing after IκBα-MnNP treatment but not the tumor weight. Overall, treatment with IκBα-MnNPs provides benefits in the context of late-stage disease but will likely need to be used in combination with other therapies or started earlier in the disease development to produce robust anti-tumor effects. These encouraging results in a late-stage model indicate a potential future avenue for evaluating the synergistic effects of combining IκBα-MnNPs with chemotherapeutics to overcome the chemoresistance often associated with late-stage ovarian tumors. Combination therapies will be necessary for late-stage tumor treatment since single therapies are unable to fully overcome the immunosuppressive TME.

The encouraging results in late-stage treatment provided insight into the effects IκBα-MnNPs have on immune cells in the TME. A second model using TBR5 ovarian tumors was adopted to examine early-stage treatments in an aggressive model. To combat the more aggressive tumor model, a biweekly treatment regimen was implemented. This treatment was also possible since the mice were not close to humane endpoint at the start of the treatment. Biodistribution was again examined in the biweekly model with a comprehensive immune cell panel to determine the efficiency of specific targeting to macrophages. These results demonstrated the preferential delivery of MnNPs to the targeted cell populations (macrophages and monocytes) that express the CD206 mannose receptor. Significant MnNP delivery to the TAM populations (89% in the ascites and 34% in the tumors) is essential for altering macrophage phenotype to overcome the immunosuppressive TME. Furthermore, the low uptake in CD45- cells, which includes tumor cells, is important for preventing off-target delivery of IκBα siRNA and therefore unwanted activation of NF-κB in cells besides macrophages. There was also minimal uptake in all other immune cells in the ascites and tumors, and less than 5% of any immune cell population in the spleen gated positive for MnNP delivery. These results indicate that the IP delivery is effectively localizing the MnNP delivery to the peritoneal cavity and increasing to the biweekly treatment does not increase the amount of nanoparticle dosage that escapes the peritoneum. Importantly, these results show improvements over previous studies using a similar NP system loaded with mRNA that included a targeting moiety, which resulted in significant off-target delivery to the spleens even after IP injections [25]. To achieve a macrophage-dependent anti-tumor immune response, it is important to repolarize a large enough population of the TAMs so that the M1 macrophages can overcome the immunosuppressive TME and increase inflammation and immune activation while also limiting potential systemic toxicity due to off-target delivery.

The therapeutic effect of IκBα-MnNP was prevalent in the TBR5 model. Importantly, increasing to a biweekly MnNP treatment did not increase toxicity based on AST, ALT, and BUN levels in the serum. These treatments also significantly decreased ascites volume, indicating an effect of the mannosylated carriers themselves, similar to the results seen in canonical NF-κB activation. The beneficial effect of mannosylated carriers alone on tumor suppression and polarizing macrophages toward the M1-like phenotype has been previously reported and is therefore not surprising in this context [25, 70]. In fact, the potential combination of mannosylated carriers with IκBα siRNA could combine for a more robust anti-tumor effect. The tumor weights were not significantly altered in any of the treatment groups, likely due to low overall tumor burden as seen by the small tumor weights, but there was a clear trend in decreasing tumor mass in the IκBα-MnNP treatment. H&E staining of the tumor sections revealed changes in tumor tissue histology and IF staining for CD8 T cells revealed a clear increase in T cell infiltration in the IκBα-MnNP treatment. This result is crucial to the future of developing macrophage-based immunotherapies as it supports the hypothesis that activating inflammatory macrophages can also “prime” solid tumors to be more responsive to T cell-based immunotherapies. This result is also supported by the significant increase in RNA expression of CXCL9 in the ascites, which is a chemokine produced by M1 macrophages to attract T cells and induce a Th1 response [71]. The evidence of increased T cell infiltration supports the potential future combination of MnNP treatment with immune checkpoint blockades which necessitate T cell infiltration for functionality. Increased tumor-infiltrating T cells and significant reduction in ascites volume associated with IκBα-MnNP treatment support the future directions of utilizing MnNPs for tumor treatments in combination therapies.

Western blot analysis of cells in the ascites revealed that the IκBα-MnNPs reduced IκBα protein levels, contributing to changes in immune cell composition. This change was also evidenced by the significant decreases in IL-6 and Arginase-1 which confirm a shift away from pro-tumor immune phenotypes. More importantly, there was a significant increase in TNF-α expression in the solid tumor. The biweekly treatment enabled MnNPs to successfully deliver to the TAMs in the solid tumor and alter immune cell phenotype. The trend in decreasing tumor weight, though not significant, demonstrated the effects of IκBα-MnNP delivery to TAMs, altering their phenotype, and inducing changes in other immune cells in the TME. The advantages of IP delivery of MnNPs to treat ovarian tumors are supported by the delivery studies and the therapeutic studies, both of which reveal nanoparticle penetration into the solid tumor as well as changes in immune cell phenotype in the tumor.

The addition of the extended treatment model was used to follow-up the original TBR5 experiment to further examine IκBα-MnNP therapeutic efficacy. The shorter treatment revealed positive signs of anti-tumor immunity, so the logical follow-up was to extend treatment by 1 week (two extra doses). These results immediately revealed the significant therapeutic benefits of MnNP delivery with the ascites volume and tumor weight being significantly reduced in the treatments. Similar to the previous cohorts, we once again saw therapeutic effects of the mannoslyated carrier alone with a more pronounced effect in the IκBα-MnNP. Furthermore, this study examined in detail the immune cell populations comprising the ascites and tumor and revealed several significant findings. The significant shift in TAMs from an M2 to M1 phenotype in vivo indicates that the IκBα-MnNPs recapitulate the results seen in vitro and can produce the inflammatory microenvironment observed through PCR results in the previous studies. This effect was seen in both the ascites and the tumors which again validates the biweekly IP injection as a therapeutically effective treatment strategy. Furthermore, these results confirm the effects of Scr-MnNPs in shift TAMs away from an M2 phenotype, and the synergistic effect of loading with IκBα siRNA is evidenced by the significant decrease in the percent of CD206+ TAMs in the IκBα-MnNP treatment compared to the Scr-MnNP treatment. Also, in line with the previous observations of increased CD8 T cell infiltration, analysis of CD8 T cell populations revealed a trend of increased infiltration in IκBα-MnNP treated mice. Further increases in inflammatory monocyte and NK cell populations confirm the ability of macrophage repolarization to recruit and effect other immune cell populations to lead to anti-tumor immune responses.

Overall, these studies indicate a therapeutic benefit from IκBα-MnNP treatment in ovarian tumors. While the late-stage treatment of ID8 tumors did not significantly alter tumor progression, there were signs of changes in the immune cell composition and a trend in decreasing ascites accumulation, indicating some positive responses. Future studies in this model may examine increasing the MnNP dosage or combining MnNP treatment with currently approved chemotherapies. Many patients develop chemoresistance in late-stage EOC and this phenomenon is often exacerbated by the buildup of ascites [19, 20]. By implementing IκBα-MnNP treatment initially to reduce ascites accumulation, we could examine the potential effects of MnNPs to restore chemotherapy responsiveness in late-stage ovarian tumors. Similarly, the positive therapeutic effects seen in the early-stage treatment of the aggressive TBR5 model, in terms of tumor progression and immune cell composition, provide context for future directions in combining MnNP treatments with T cell-targeted immunotherapies. Many of the current T cell-based therapies are not as effective in treating solid tumors due to low T cell infiltration and high levels of immunosuppressive cytokines. Targeting TAMs to produce pro-inflammatory cytokines, such as IFN-γ and TNF-α, can promote an immune-favorable microenvironment while also producing T cell-specific chemokines, such as CXCL9, to recruit T cells to the tumor. The logical next step in this research will be to examine combination treatments with ICBs, such as anti-PD-1 s, to potentially produce a more robust immune response and develop immunological memory to prevent subsequent recurrence. The results of this study demonstrated positive therapeutic results but have also informed our future directions for developing therapies to treat ovarian cancer in patients.

## Conclusions

This work demonstrates the efficacy of MnNPs in inducing TAM-mediated anti-tumor immunity in mouse models of ovarian cancer. Delivery of IκBα siRNA to NGL-BMDMs activated canonical NF-κB, indicating repolarization of the macrophages toward an inflammatory M1-like phenotype. Biodistribution studies in two different tumor models revealed preferential MnNP association with TAM and myeloid cell populations only in the ascites and solid tumors with minimal off-target delivery to other immune cells and negligible uptake in any cells in the spleens. These results supported the use of IP injections to provide localized delivery to the TME. The delivery of MnNPs to macrophages in the ascites led to a slight decrease in ascites buildup in the late-stage model, but a substantial decrease in the aggressive TBR5 model. The positive effect of mannosylated carriers on preventing ascites development in the aggressive TBR5 model indicates a potential for synergistic effects of IκBα siRNA with MnNPs. While the late-stage treatment of ID8 tumors did not significantly alter tumor progression, there were noticeable differences in RNA expression of various M1- and M2-associated markers, indicating beneficial immunostimulatory changes in the TME after MnNP treatment. In the more aggressive TBR5 model, the IκBα-MnNP treatment led to slight decreases in tumor weight, but also was associated with larger numbers of infiltrating CD8 T cells. The increase in tumor-associated T cells is highly encouraging for future studies involving combination treatments with immune checkpoint blockades which rely on the presence of T cells in the tumors for anti-cancer toxicity. Additionally, we demonstrated that an extended treatment of MnNPs in the TBR5 model significantly suppressed ascites accumulation and tumor development while also altering the immune cell composition in the TME. These results further support the hypothesis that activating macrophages can transform the TME into a pro-inflammatory niche which will support ICB therapies. Importantly, this study shines a light on the potential for IP administered therapies that can influence tumors in the peritoneal cavity through modulation of cells in the ascites. Overall, this work highlights the use of MnNPs in IP treatment of ovarian cancer and provides a pathway forward for enhancing the current treatment paradigm in patients with a highly deadly disease.

## Supplementary Information

**Additional file 1.**

## Data Availability

The datasets used and/or analyzed during the current study are available from the corresponding author on reasonable request.

## Abbreviations

ALT: Alanine transaminase
ANOVA: Analysis of variance
AST: Aspartate transaminase
Az: Azide
BMA: Butyl methacrylate
BMDM: Bone marrow-derived macrophage
BSA: Bovine serum albumin
BUN: Blood urea nitrogen
CCL3: C-C motif ligand 3
CTA: Chain transfer agent
CuAAC: Copper-catalyzed azide-alkyne cycloaddition
CXCL9: C-X-C motif ligand 9
Cy5: Cyanine-5
DAPI: 4’,6-diamidino-2-phenylindole
DMAEMA: Dimethylaminoethyl methacrylate
DMEM: Dulbecco’s modified eagle medium
dsDNA: Double-stranded deoxyribonucleic acid
ECT: 4-cyano-4-(ethylsulfanylthiocarbonyl)-sulfanylpentanoic acid
EOC: Epithelial ovarian cancer
EtOH: Ethanol
FACS: Fluorescence-activated cell sorting
FBS: Fetal bovine serum
FDA: Food and Drug Administration
FSC: Forward scatter
FTIR: Fourier transform infrared
FVB: Friend leukemia virus B
GFP: Green fluorescent protein
H&E: Hematoxylin and eosin
IACUC: Institutional Animal Care and Use Committee
ICB: Immune checkpoint blockade
IF: Immunofluorescence
IFN-γ: Interferon-gamma
IgG: Immunoglobulin G
IκBα: Inhibitor of NF-kappaB alpha
IL-4: Interleukin-4
IL-6: Interleukin-6
IP: Intraperitoneal
IRF-5: Interferon regulatory factor 5
IV: Intravenous
LPS: Lipopolysaccharide
MnNP: Mannosylated nanoparticle
MWCO: Molecular weight cutoff
NF-κB: Nuclear factor-kappaB
NGL: NF-κB green fluorescent protein-luciferase
NMR: Nuclear magnetic resonance
ORR: Overall response rate
PBS: Phosphate buffered saline
PEG: Poly (ethylene glycol)
PRR: Pattern recognition receptor
P/S: Penicillin-streptomycin
qRT-PCR: Quantitative reverse transcription polymerase chain reaction
RAFT: Reversible addition-fragmentation chain-transfer
RBC: Red blood cell
RNA: Ribonucleic acid
RNAi: RNA interference
RT: Room temperature
Scr: Scrambled
siRNA: Small interfering RNA
SSC: Side scatter
TAM: Tumor-associated macrophage
TBS: Tris-buffered saline
TBST: Tris-buffered saline + Tween 20
TME: Tumor microenvironment
TNBC: Triple negative breast cancer
TNF-α: Tumor necrosis factor-alpha
TPSR: Translational Pathology Shared Resource
VINSE: Vanderbilt Institute of Nanoscale Science and Engineering
VU: Vanderbilt University
VUMC: Vanderbilt University Medical Center
