# Supplementary material for: Stimulating TAM-mediated anti-tumor immunity with mannose-decorated nanoparticles in ovarian cancer
Source: BMC Cancer. 2022 May 6;22:497. doi: 10.1186/s12885-022-09612-2 (PMC9074180; doi:10.1186/s12885-022-09612-2)

**Supplemental Table 1:** Oligonucleotide sequences used for MnNP loading.

| **Oligonucleotide** | **Sense Strand (5’🡪3’)** | **Antisense Strand (5’🡪3’)** |
| --- | --- | --- |
| Cy5 dsDNA | dGdTdC dAdGdA dAdAdT dAdGdA dAdAdC dTdGdG dTdCdA dTdC | [Cy5]-dGdAdT dGdAdC dCdAdG dTdTdT dCdTdA dTdTdT dCdTdG dAdC |
| Scr siRNA | rArUrC rUrArG rGrCrC rGrCrU rArUrA rCrCrA rArGrU | rArCrU rUrGrG rUrArU rArGrC rGrGrC rCrUrA rGrArU |
| IκBα siRNA | rGrUrA rGrCrA rGrUrC rUrUrG rArCrG rCrArG rAdTdT | rUrCrU rGrCrG rUrCrA rArGrA rCrUrG rCrUrA rCdAdC |
| Key: d = DNA base, r = RNA base  All oligonucleotides were purchased from Integrated DNA Technologies | | |


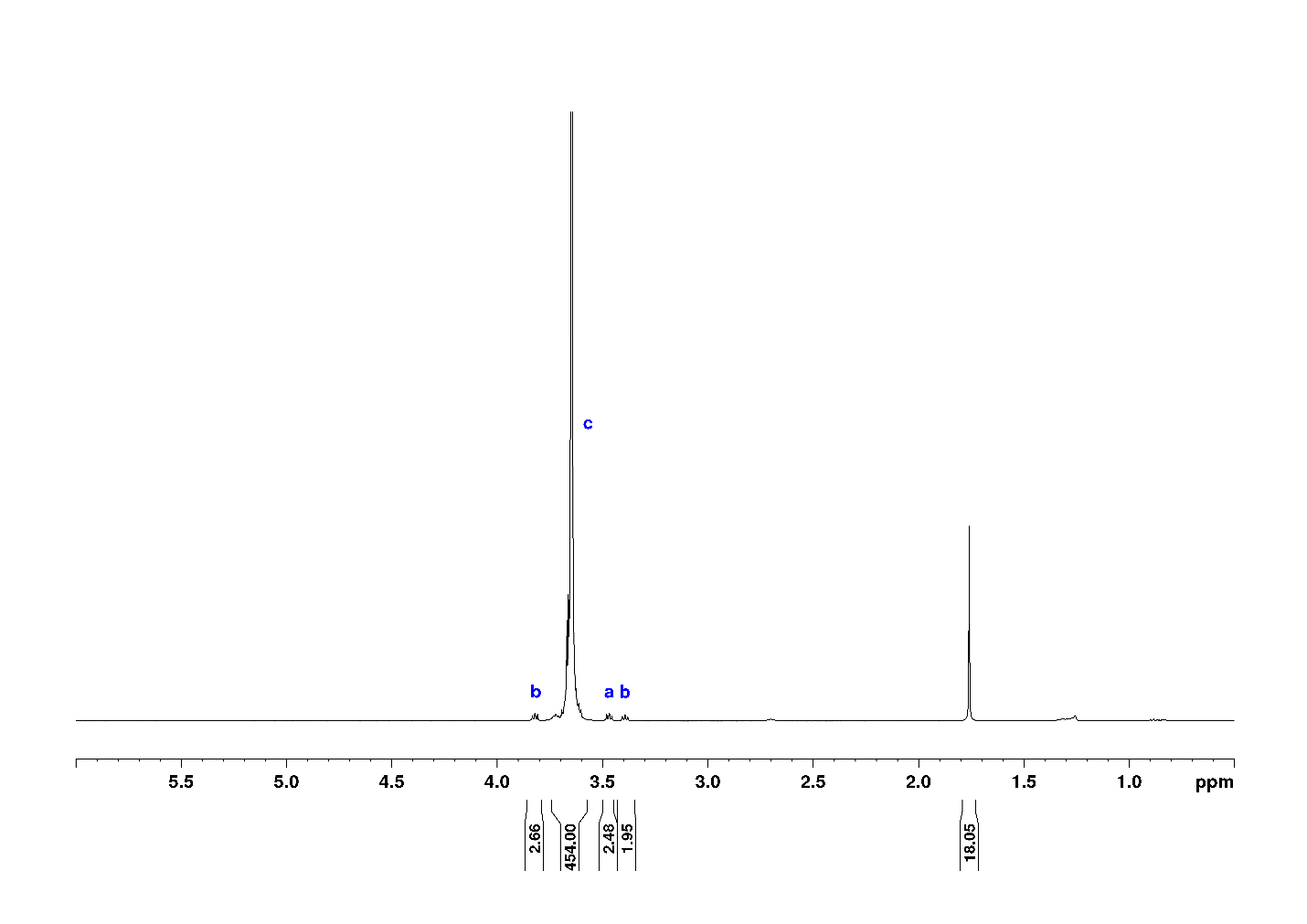


**Supplemental Figure S1:** ^1^H-NMR of azide-PEG-hydroxyl in CDCl_3_. All peaks were normalized to the PEG (5 kDa) peak at δ 3.65 (-OC**H**_2_C**H**_2_-).


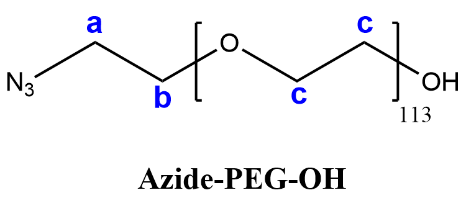

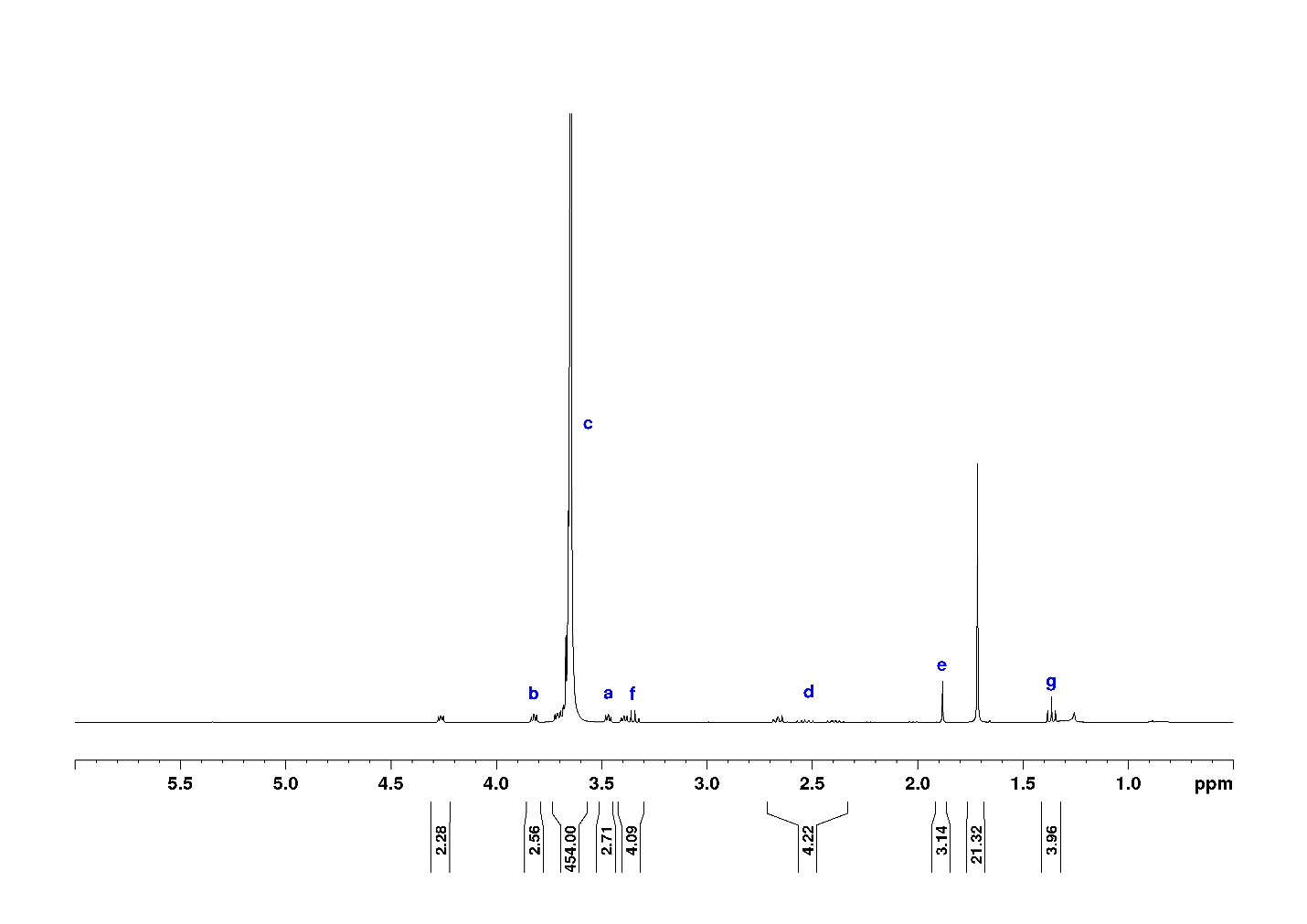

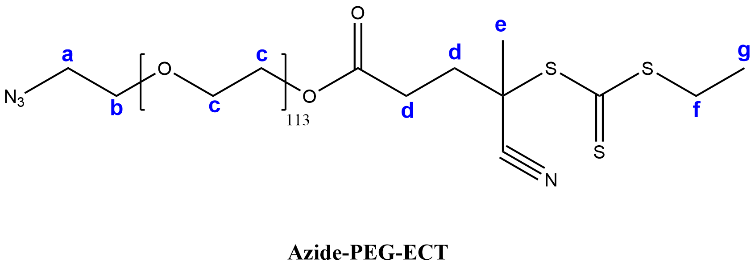


**Supplemental Figure S2:** ^1^H-NMR of azide-PEG-ECT in CDCl_3_. Formation of the macro-CTA was confirmed by the characteristic ECT peak at δ 1.88 (CCNC**H**_3_).


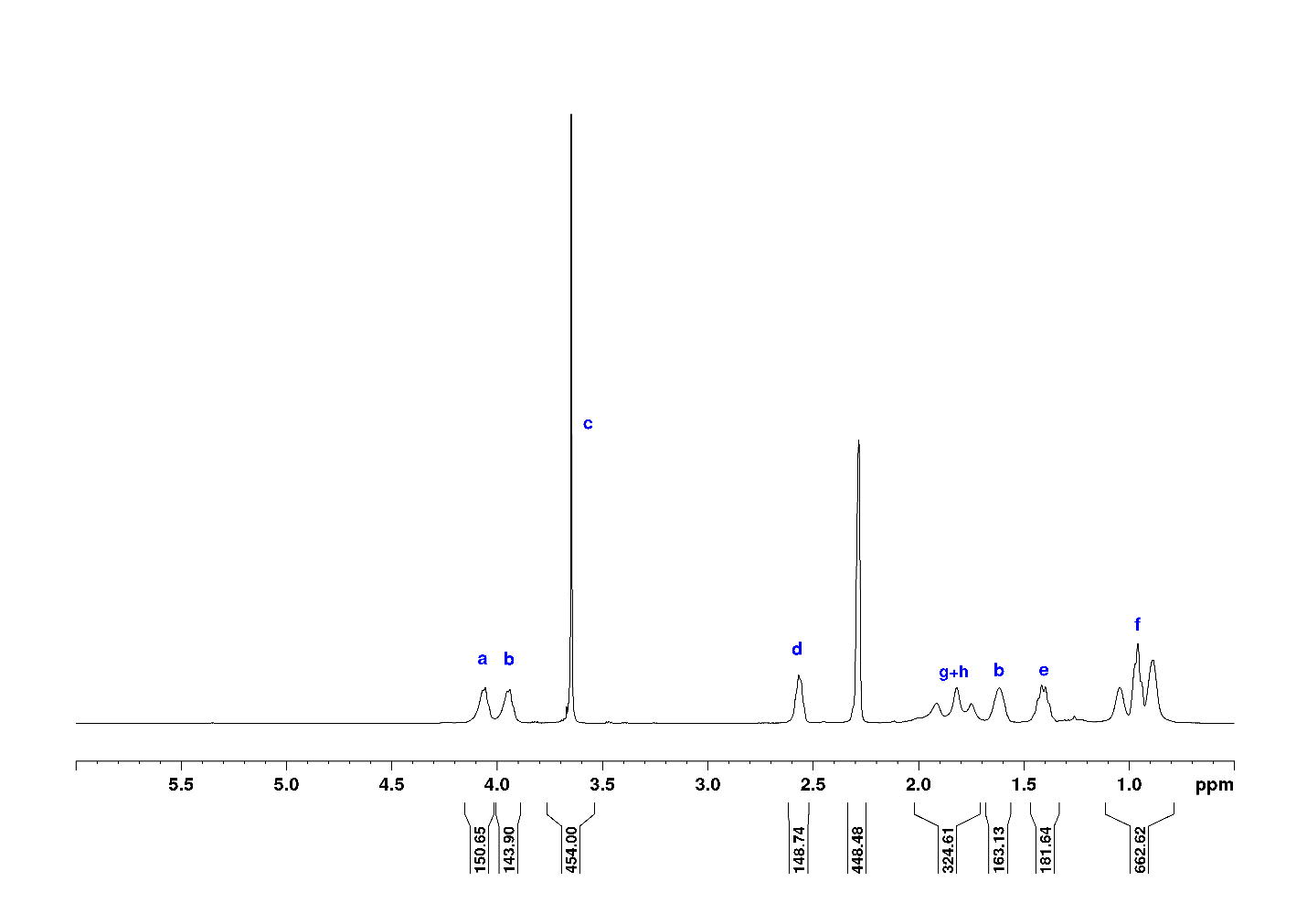

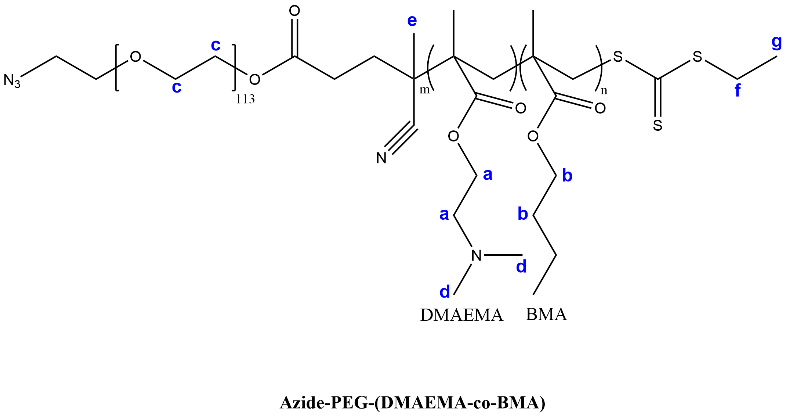


**Supplemental Figure S3:** ^1^H-NMR of azide-PEG-(DMAEMA-co-BMA) in CDCl_3_. RAFT polymerization of the diblock copolymer was confirmed by the formation of the DMAEMA peak at δ 4.05 (-OC**H**_2_C**H**_2_-) and the BMA peak at δ 3.95 (-OC**H**_2_C**H**_2_-). Number of repeating units for DMAEMA and BMA were calculated by calibrating to the PEG peak of a known length.


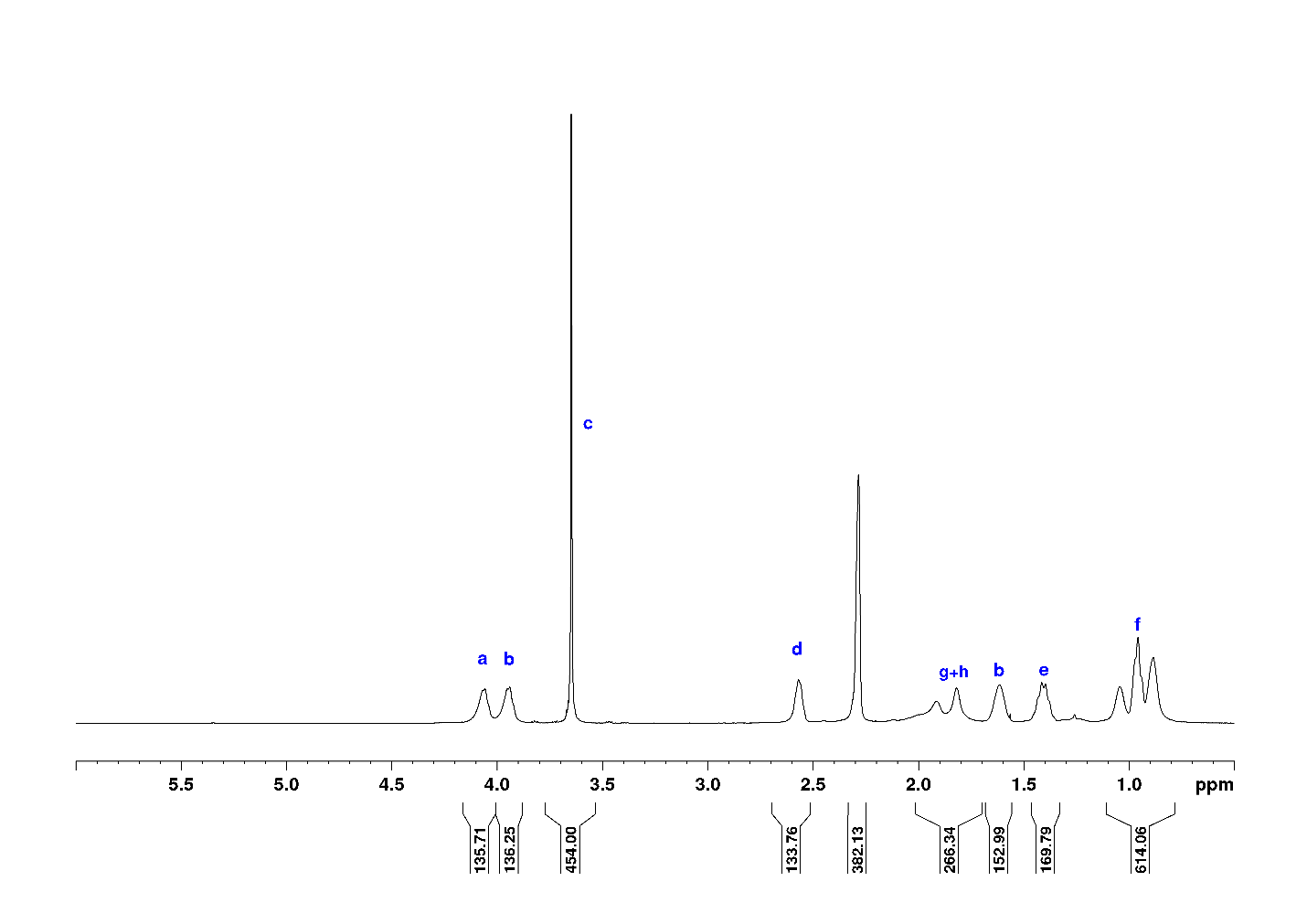

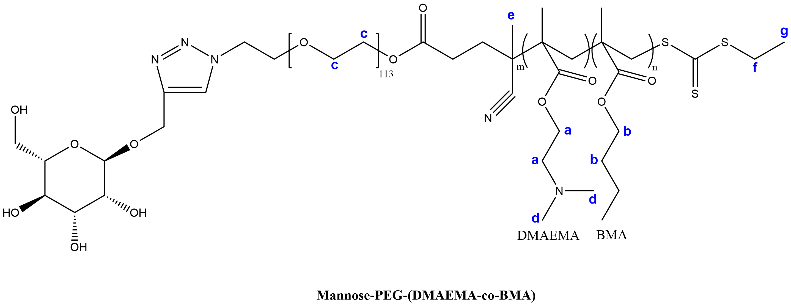


**Supplemental Figure S4:** ^1^H-NMR of mannose-PEG-(DMAEMA-co-BMA) in CDCl_3_. Mannose conjugation cannot be ascertained via NMR due to the overlap of the mannose ^1^H-NMR spectra (3.5-4 ppm) with the PEG peak.


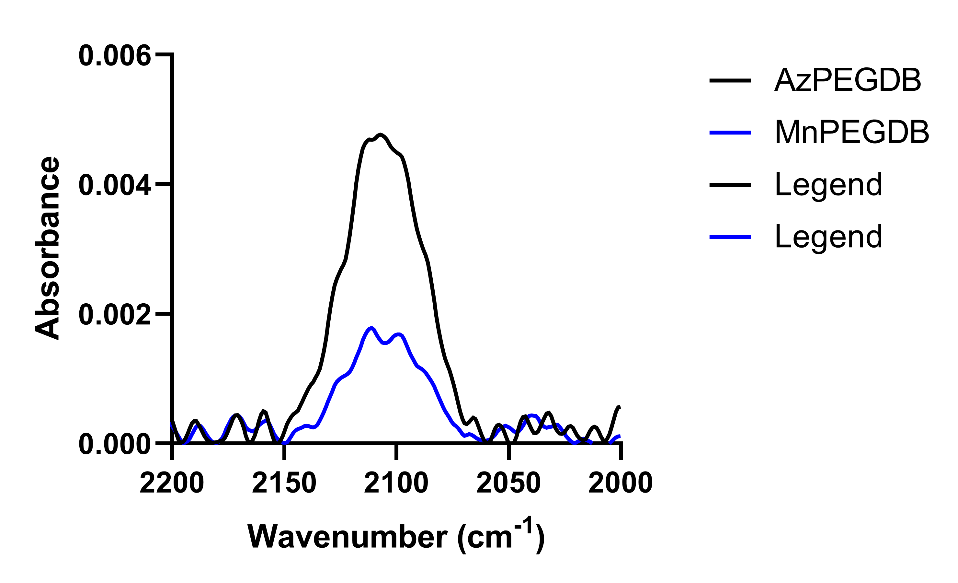

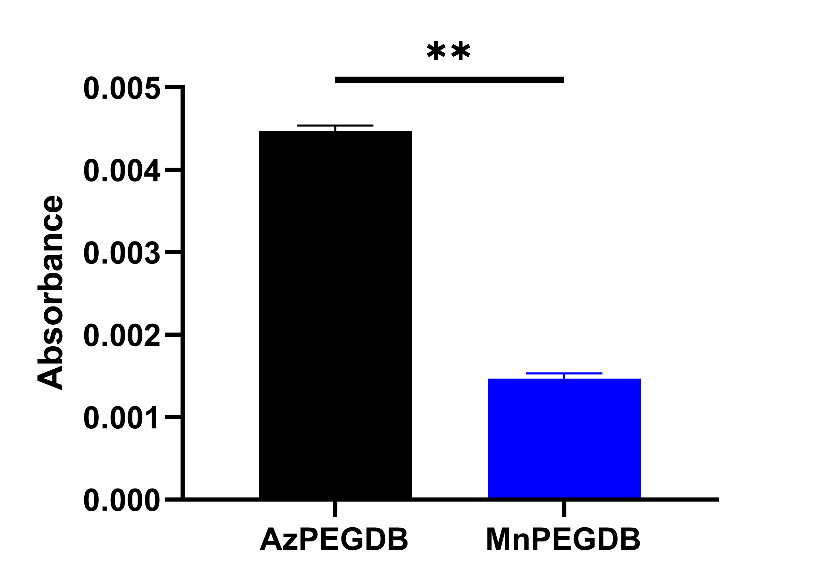

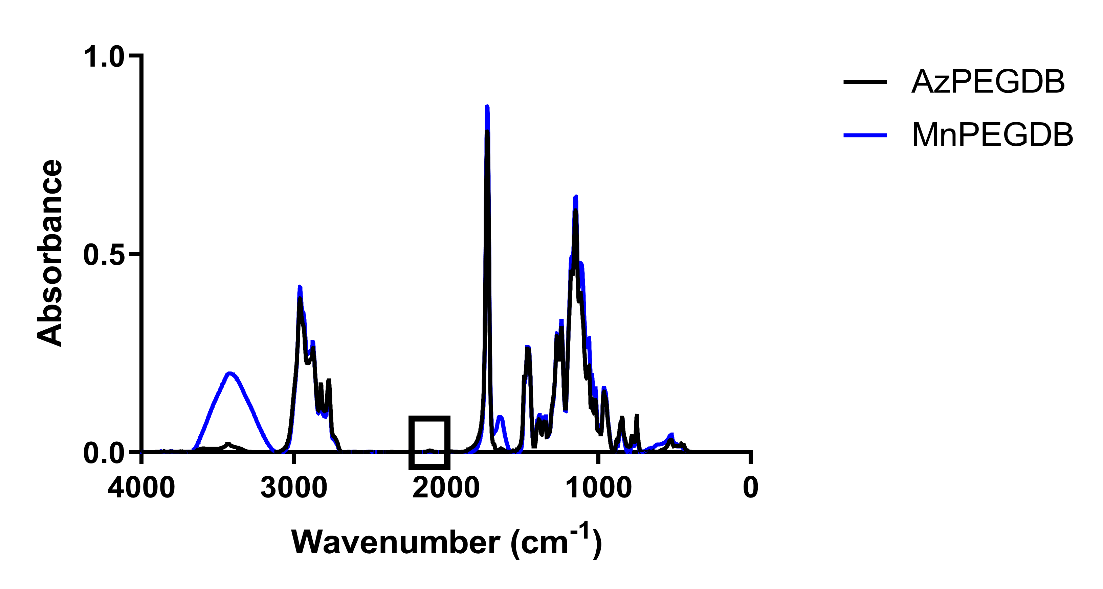

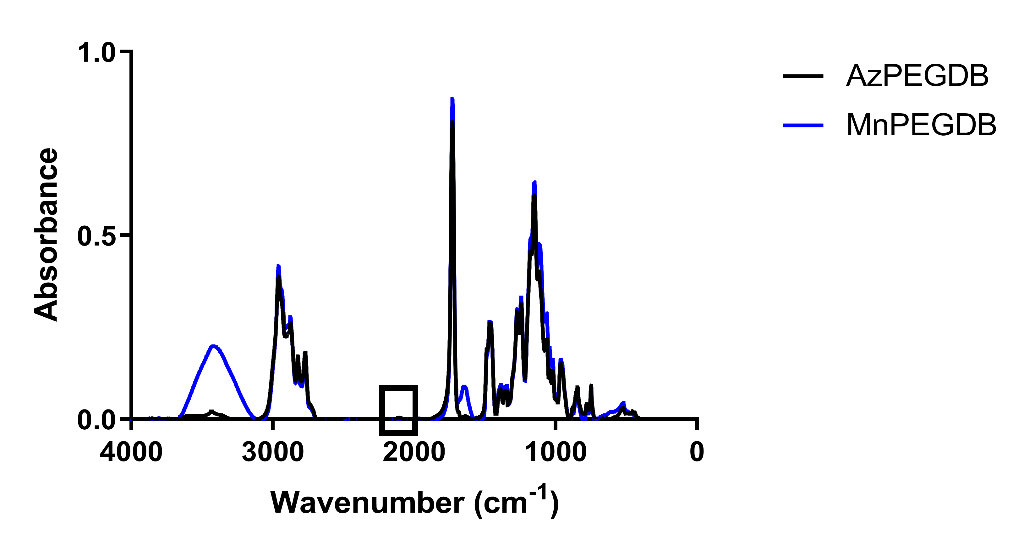


**Supplemental Figure S5:** FTIR spectroscopy of AzPEGDB and MnPEGDB to quantify mannose conjugation. (**A**) Overall FTIR spectra reveals identical spectra with an increase in absorbance around 3300-3400 cm^-1^ consistent with the FTIR spectra of d-mannose. (**B**) Detailed view of the characteristic azide peak at 2100 cm^-1^ and (**C**) quantified azide peak height revealing a significant decrease in azide presence on the MnPEGDB (**p<0.001).

A

B

C


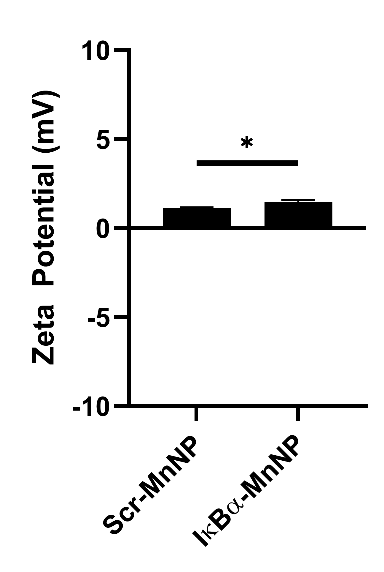

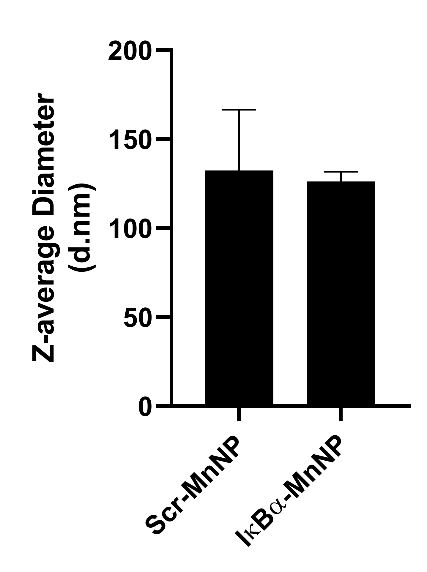


**Supplemental Figure S6:** Polyplexes were formulated with either scrambled or IκBα siRNA and suspended in PBS (-/-, pH=7.4). (**A**) Z-average diameter averaged 130 nm for both formulations. (**B**) The zeta potentials for each were below 1.5 mV, indicating a near-neutral surface charge (*p<0.05).

A

B

**Supplemental Figure S7:** Gating strategy for flow cytometry of ID8 ovarian tumor model with single 24-hr MnNP delivery. This panel included CD45 for immune cells, F4/80 for macrophages, and Gr-1 for distinguishing mature macrophages (Gr-1-) vs myeloid cells (Gr-1+). Gating is shown for tumors, ascites, and spleens.


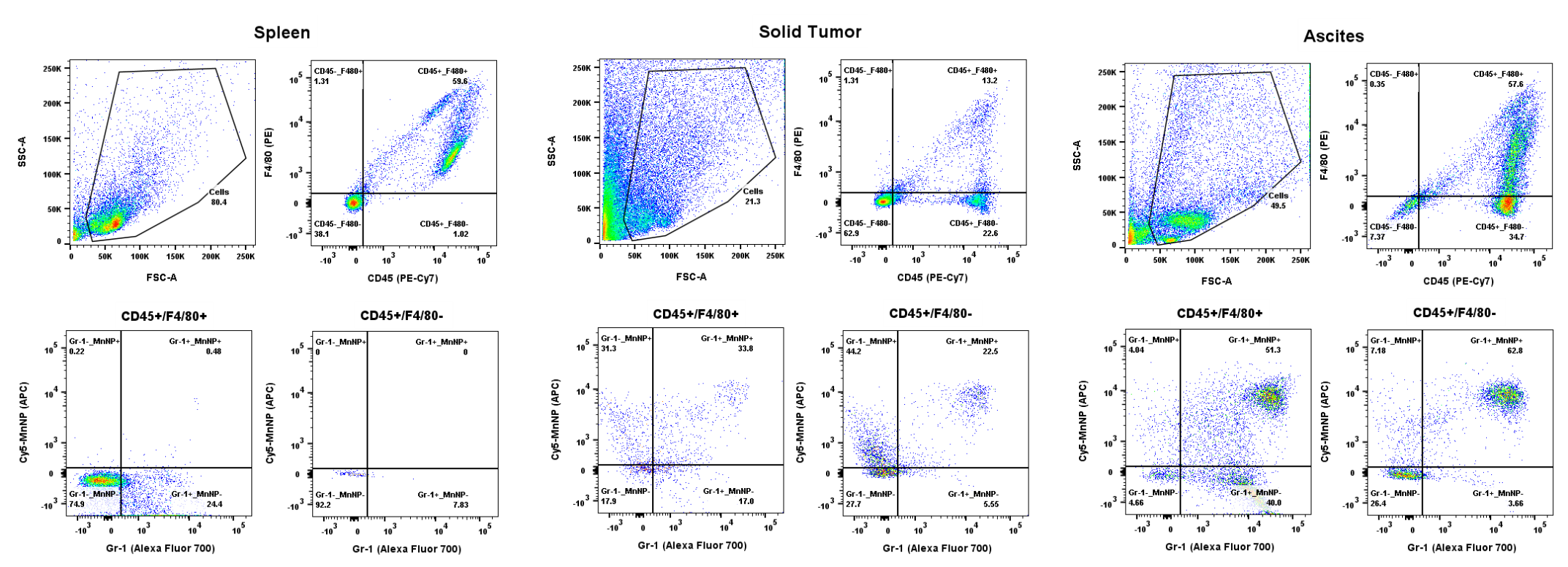

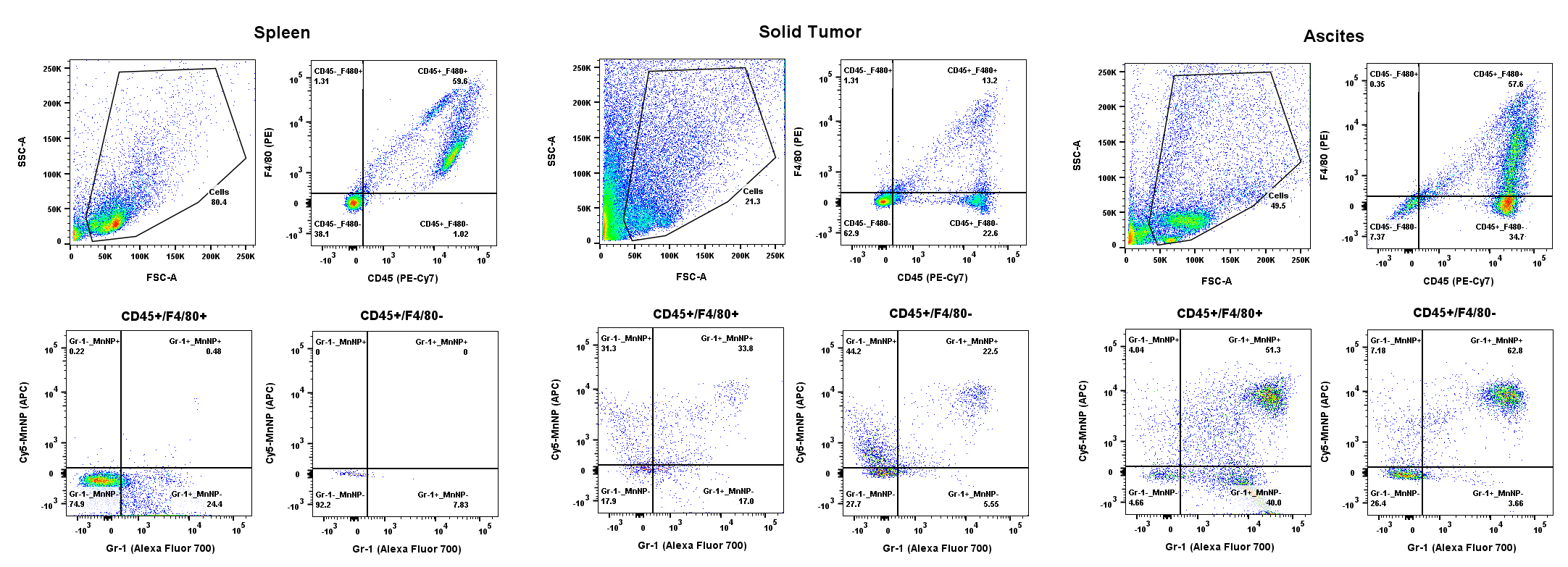


**Supplemental Figure S8:** Gating strategy for flow cytometry of solid tumors in the TBR5 ovarian tumor model with biweekly MnNP delivery. The initial gating was performed using markers for live/dead (Ghost Dye Red) and pan-immune cells (CD45). The lymphocyte panel used markers for T cells (CD3, CD4, and CD8a), Natural Killer cells (NKp46), and B cells (B220). The myeloid panel used markers for dendritic cells (CD11c), macrophages (CD11b and F4/80), and myeloid cells (Ly6C and Ly6G).


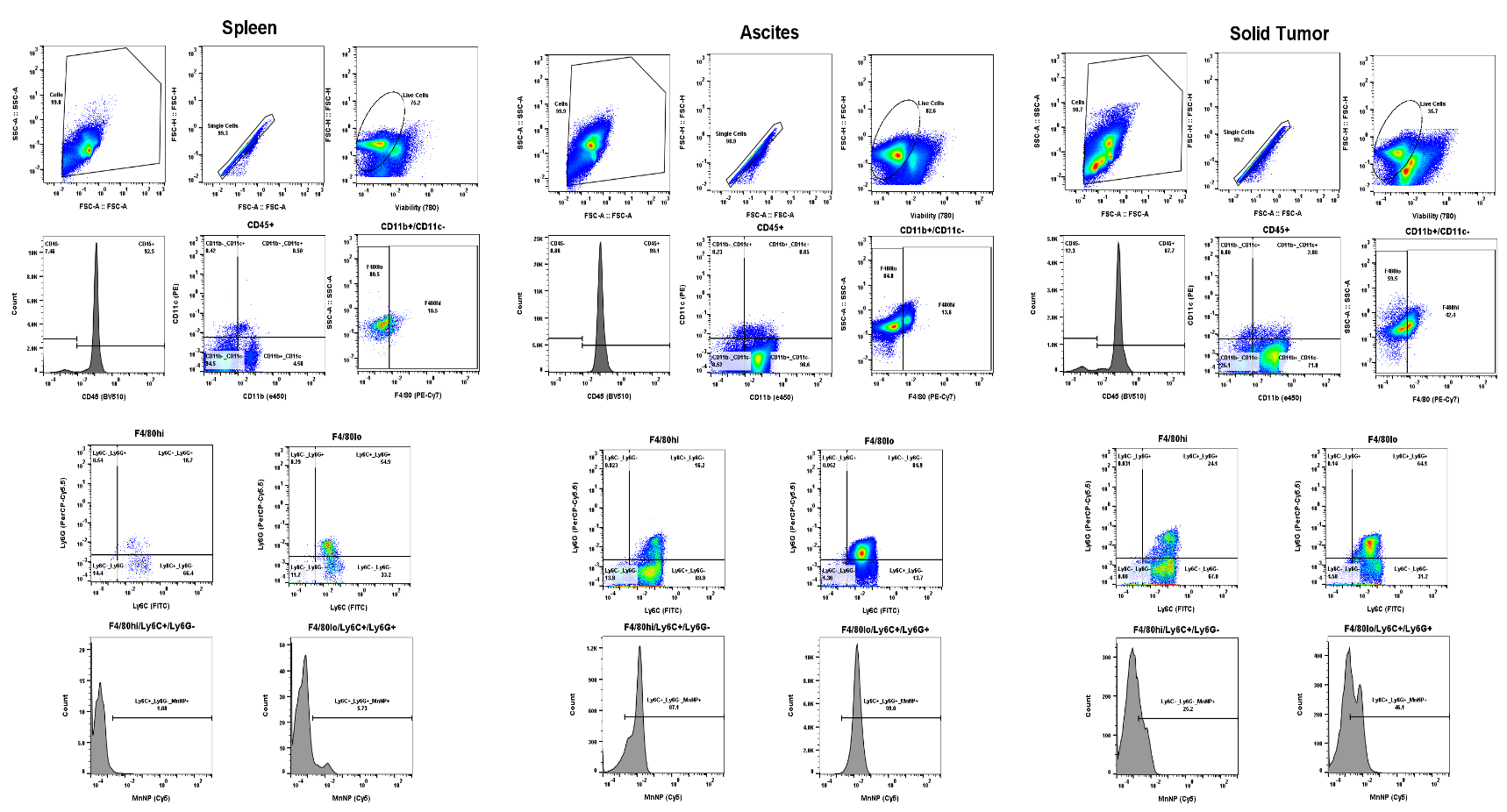

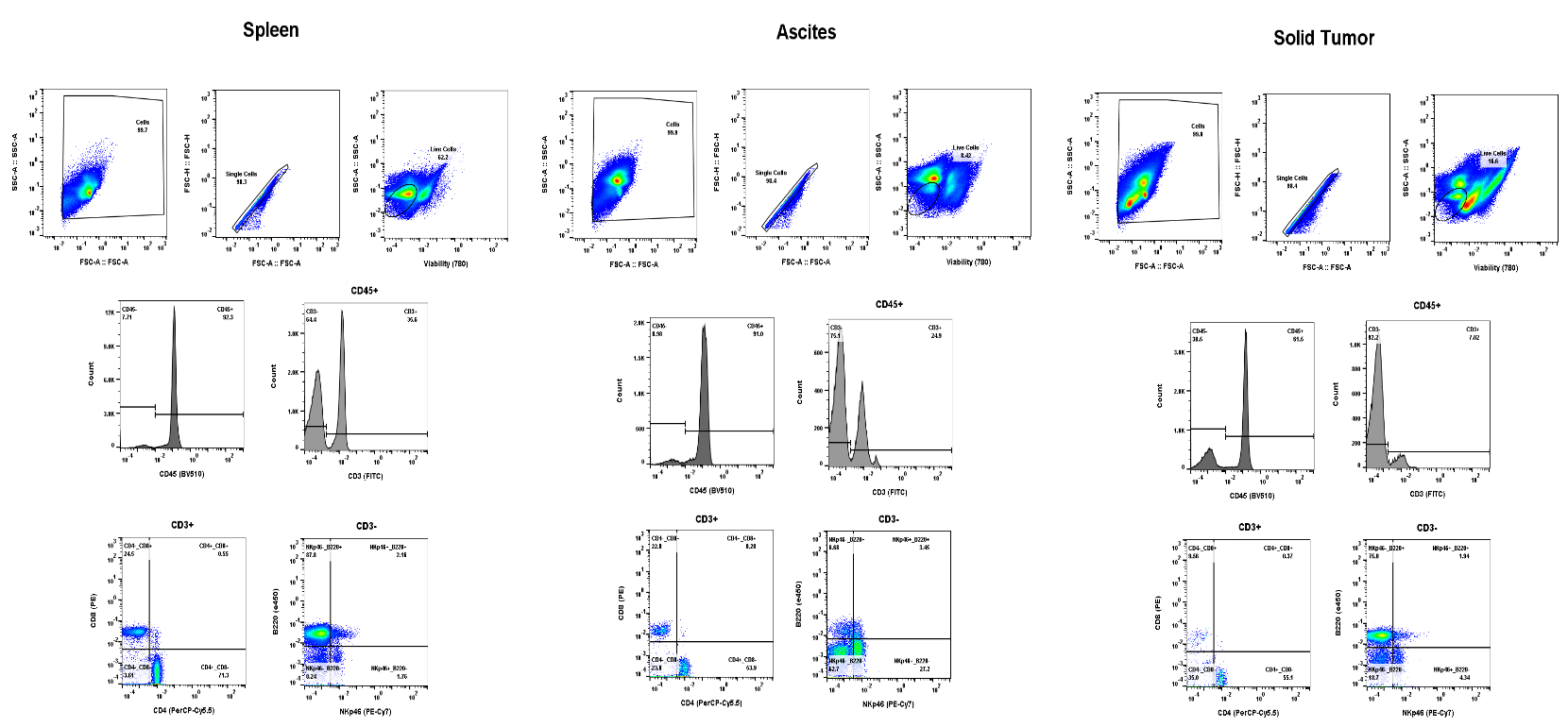

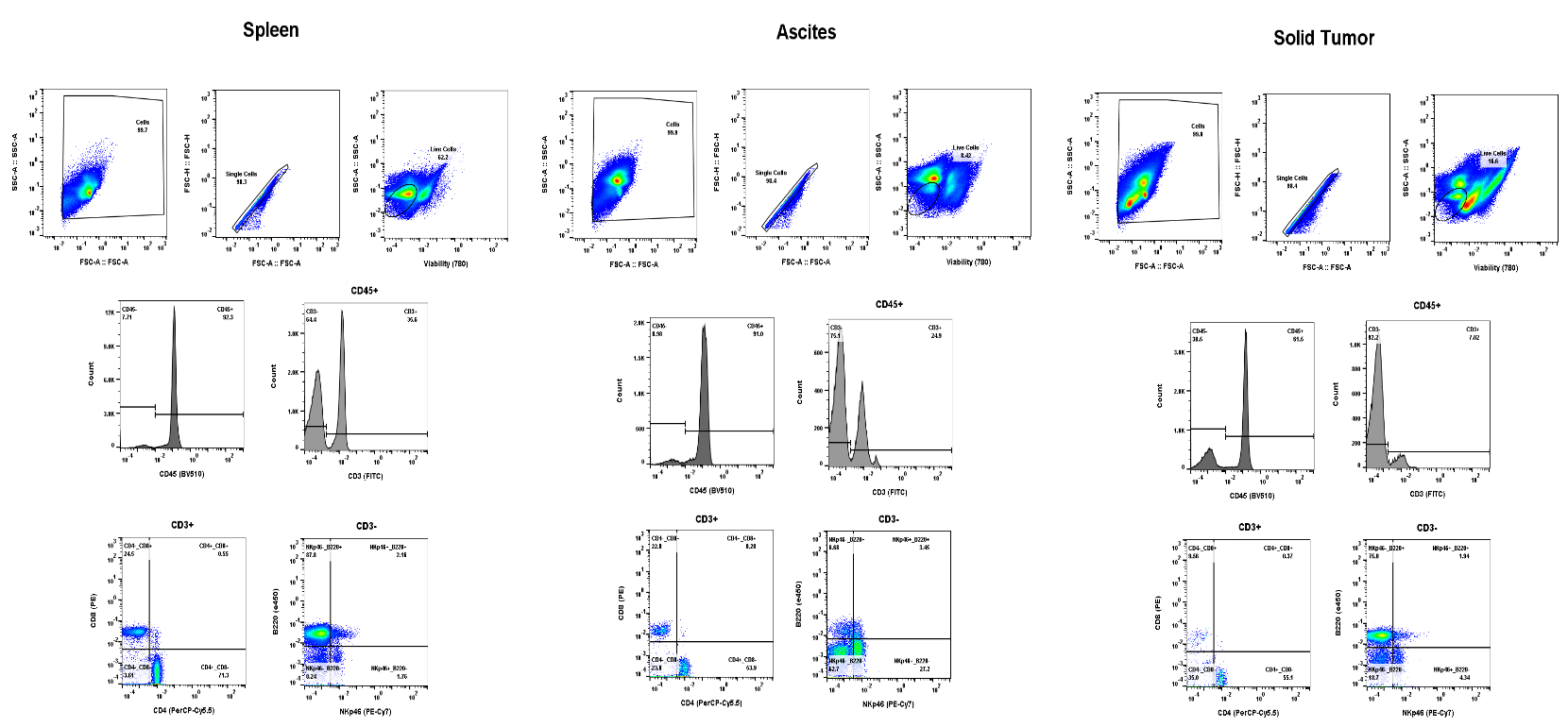


**Lymphocyte Panel**

**Myeloid Panel**

**Supplemental Figure S9:** Gating strategy for flow cytometry of ascites cells in the TBR5 ovarian tumor model with biweekly MnNP delivery. The initial gating was performed using markers for live/dead (Ghost Dye Red) and pan-immune cells (CD45). The lymphocyte panel used markers for T cells (CD3, CD4, and CD8a), Natural Killer cells (NKp46), and B cells (B220). The myeloid panel used markers for dendritic cells (CD11c), macrophages (CD11b and F4/80), and myeloid cells (Ly6C and Ly6G).


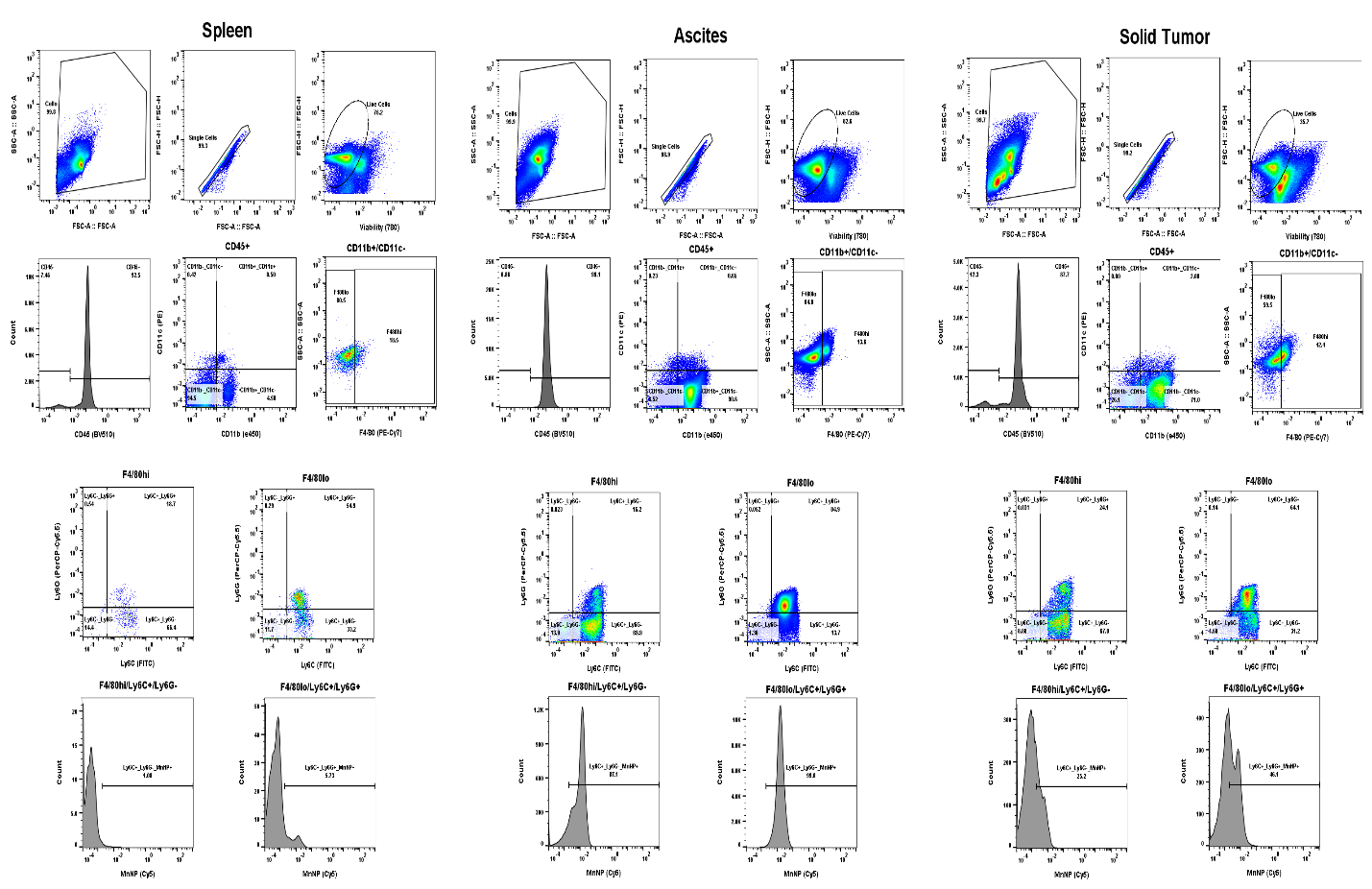

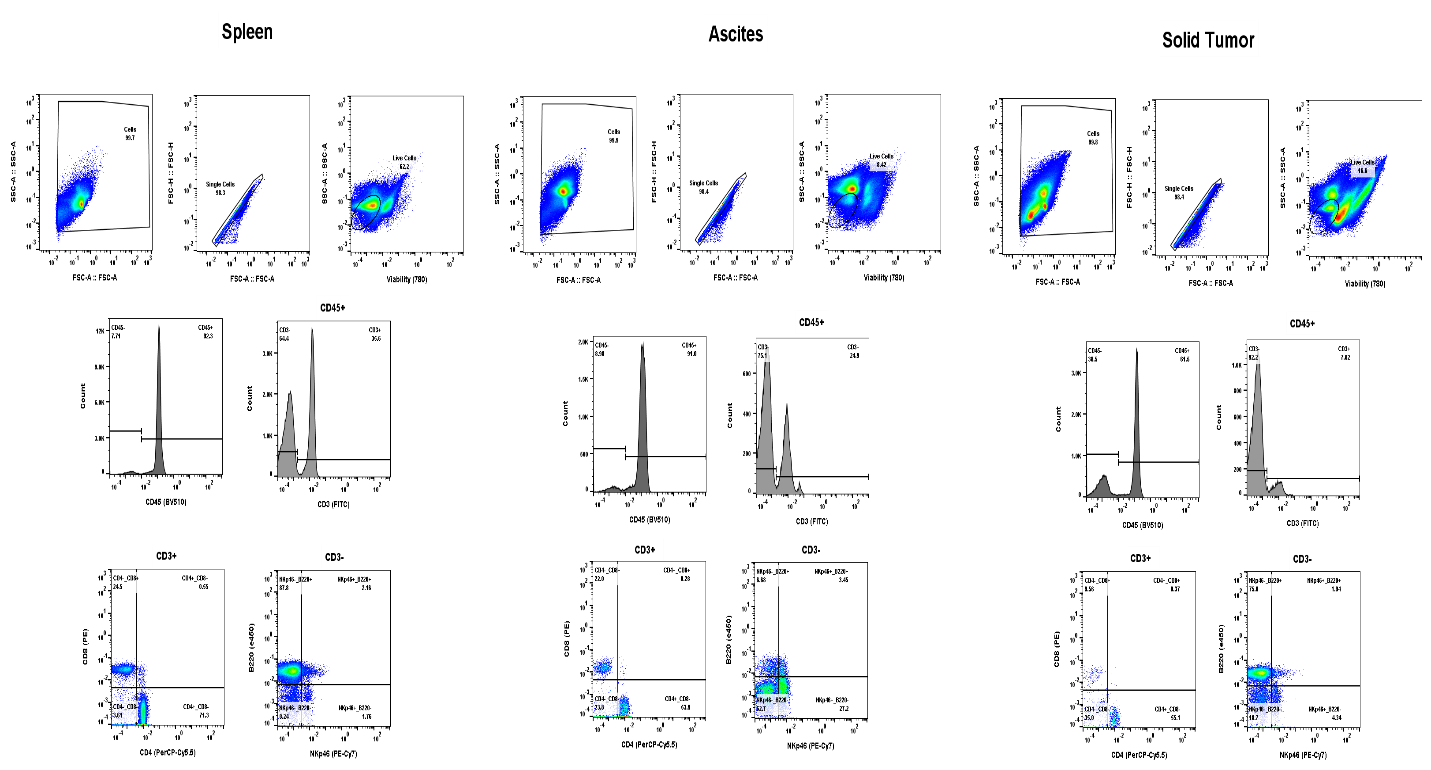

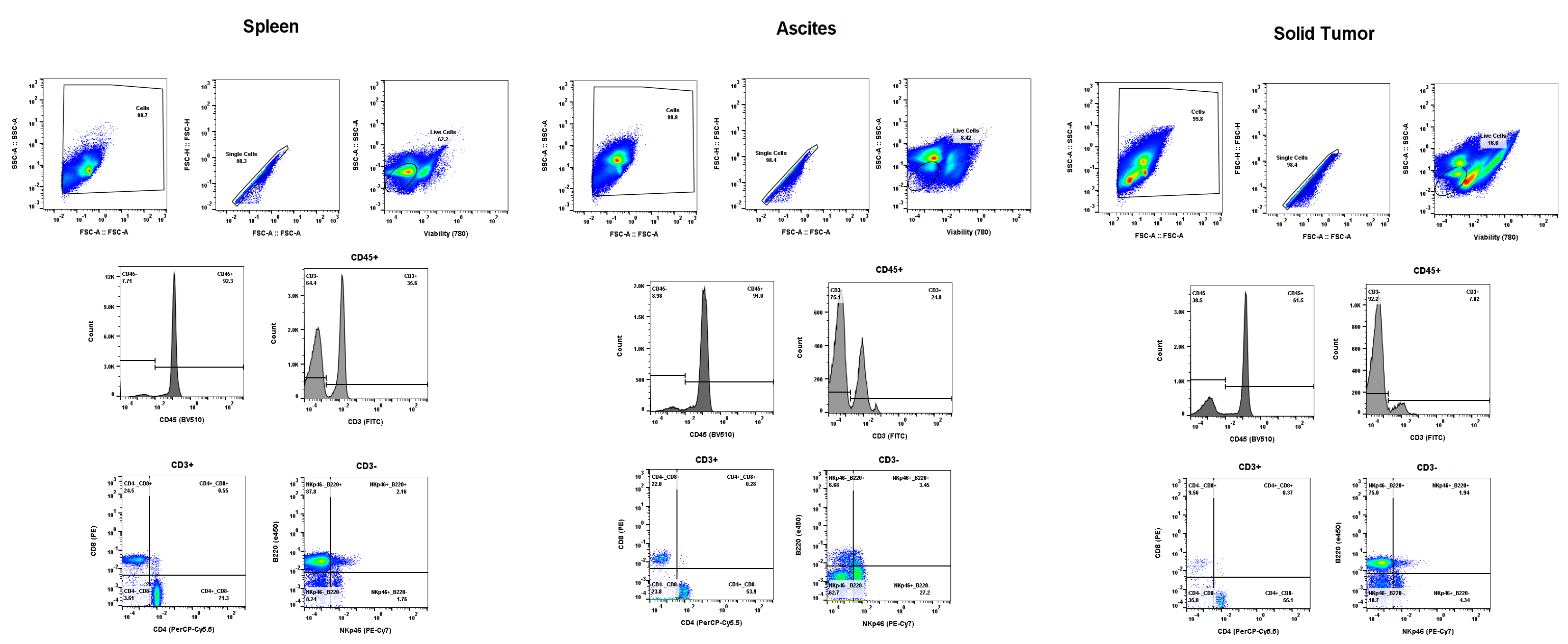


**Lymphocyte Panel**

**Myeloid Panel**

**Supplemental Figure S10:** Gating strategy for flow cytometry of spleens in the TBR5 ovarian tumor model with biweekly MnNP delivery. The initial gating was performed using markers for live/dead (Ghost Dye Red) and pan-immune cells (CD45). The lymphocyte panel used markers for T cells (CD3, CD4, and CD8a), Natural Killer cells (NKp46), and B cells (B220). The myeloid panel used markers for dendritic cells (CD11c), macrophages (CD11b and F4/80), and myeloid cells (Ly6C and Ly6G).


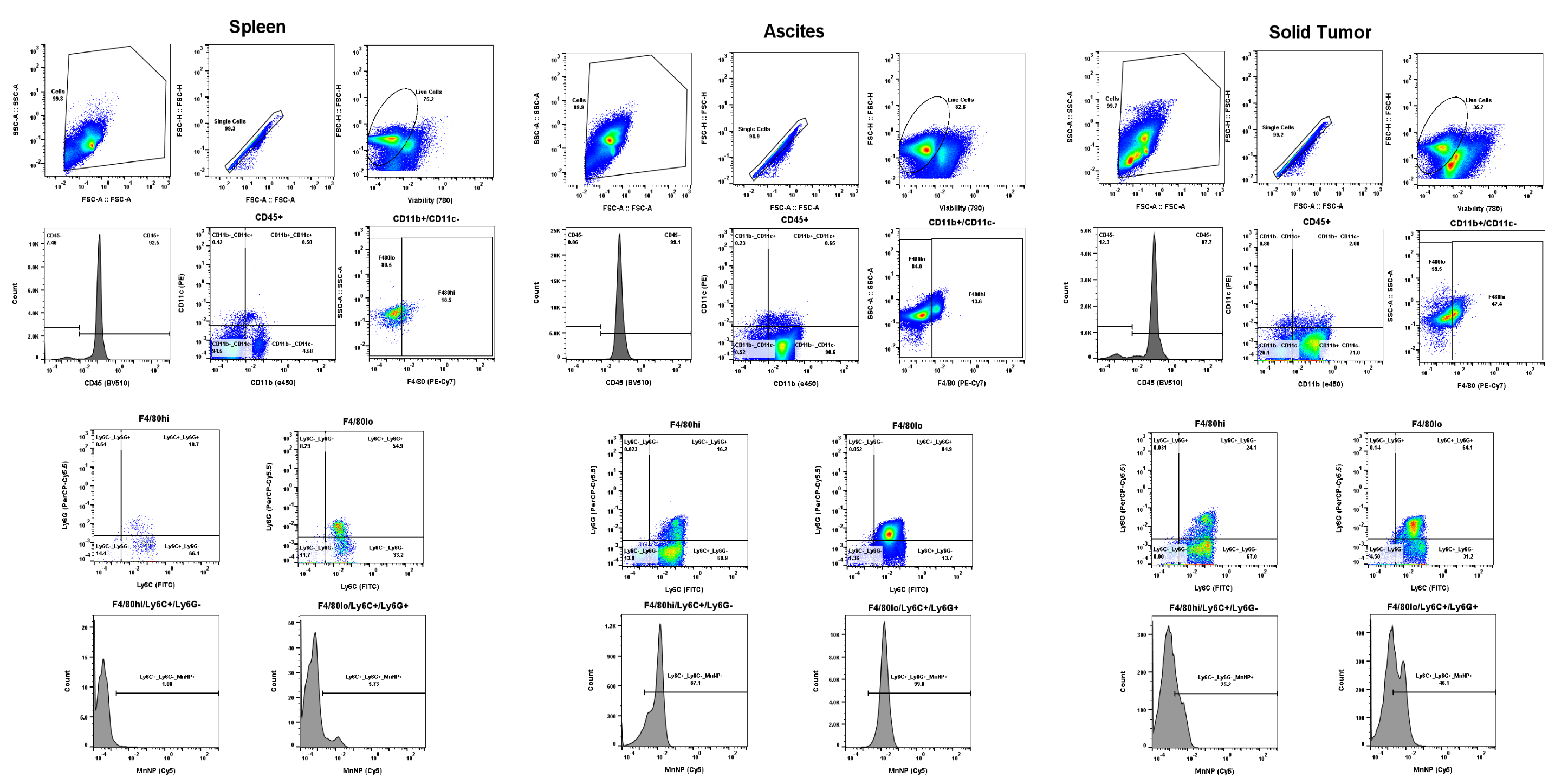

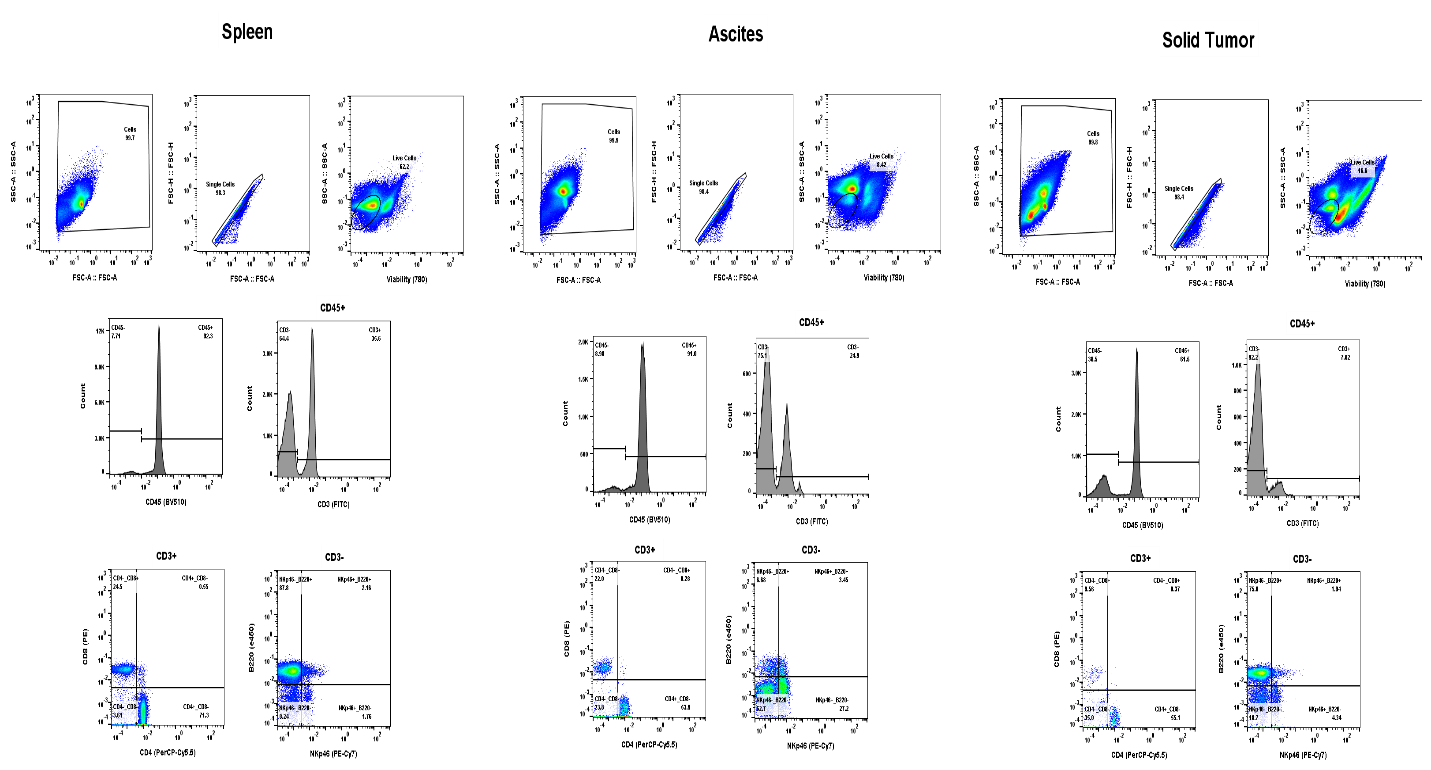

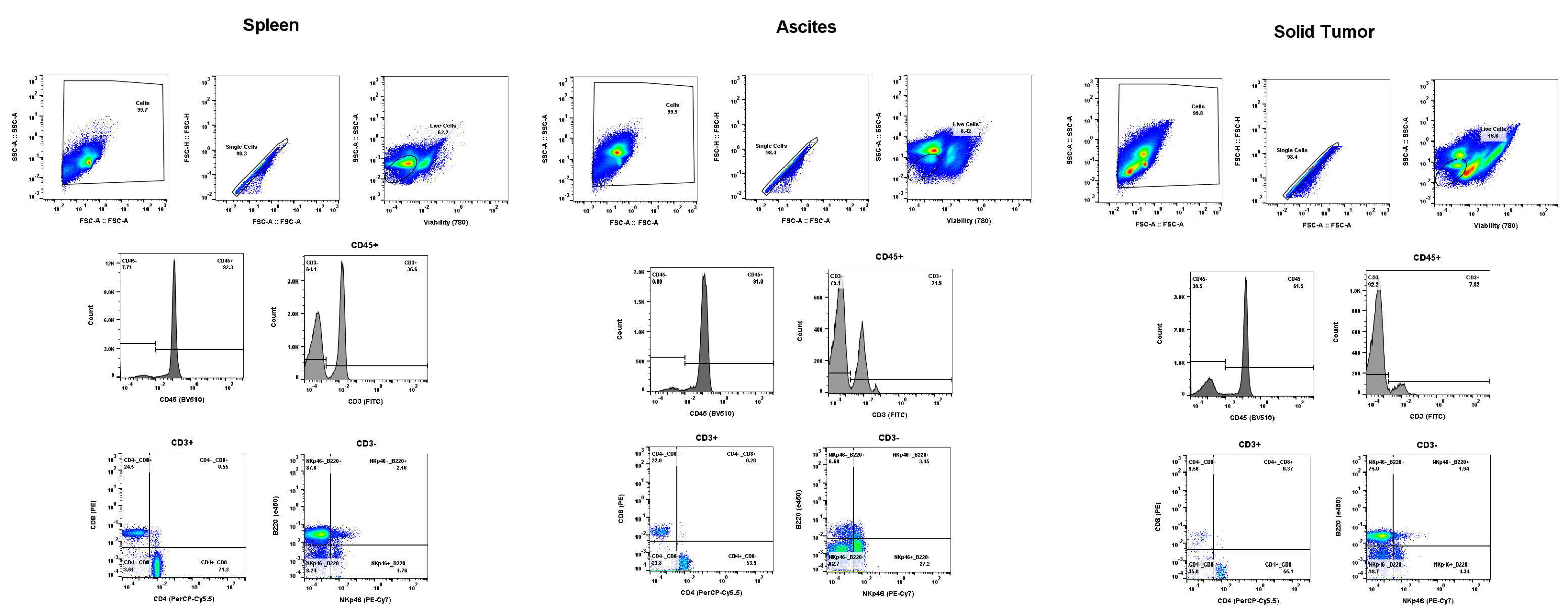


**Lymphocyte Panel**

**Myeloid Panel**

**Supplemental Figure S11:** Gating strategy for quantifying tumor cells populations. Tumor cells were gated as CD45-/SSChi (Big), with the large cell population only present in the tumors and ascites samples and not the spleen.


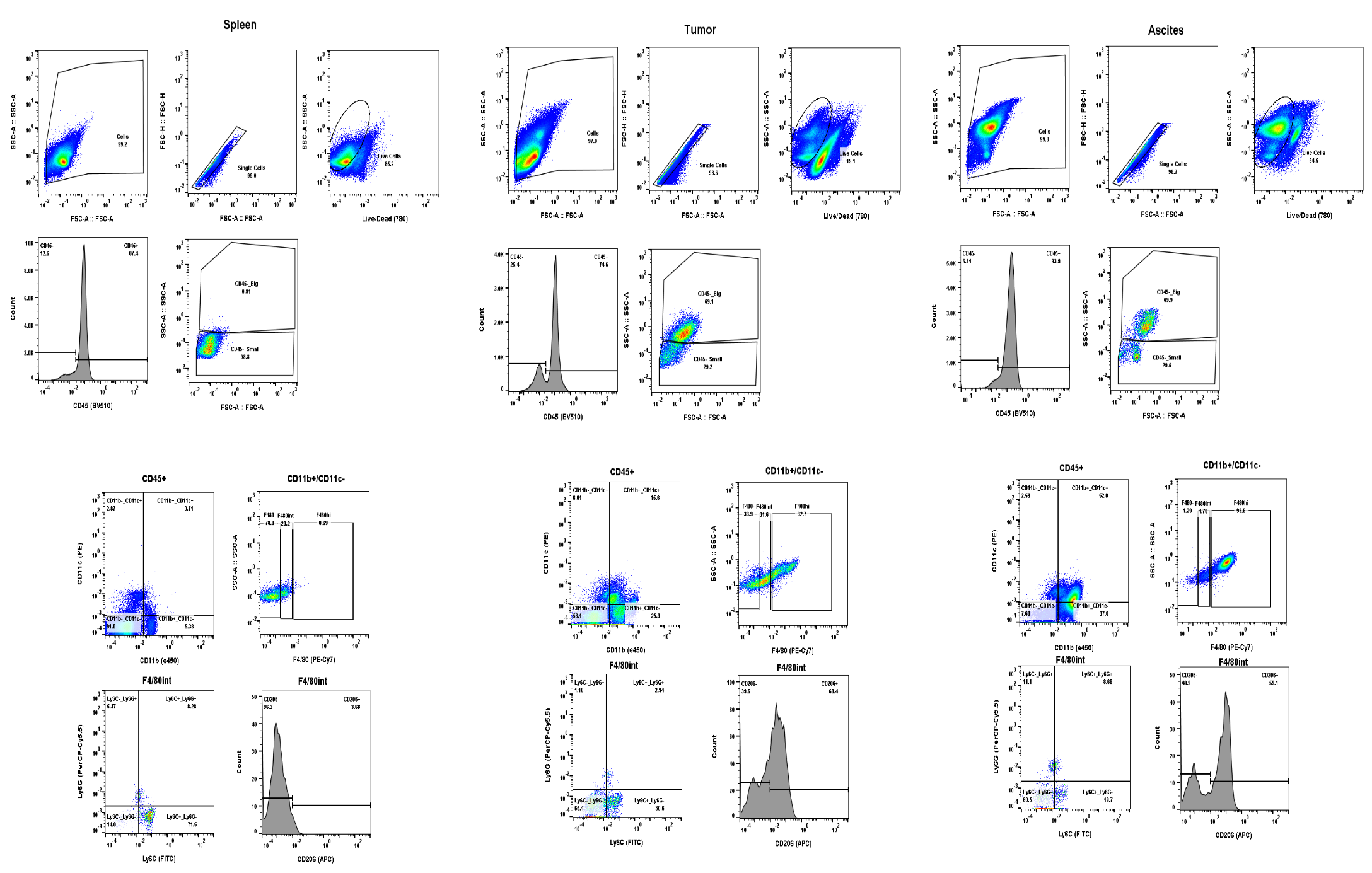

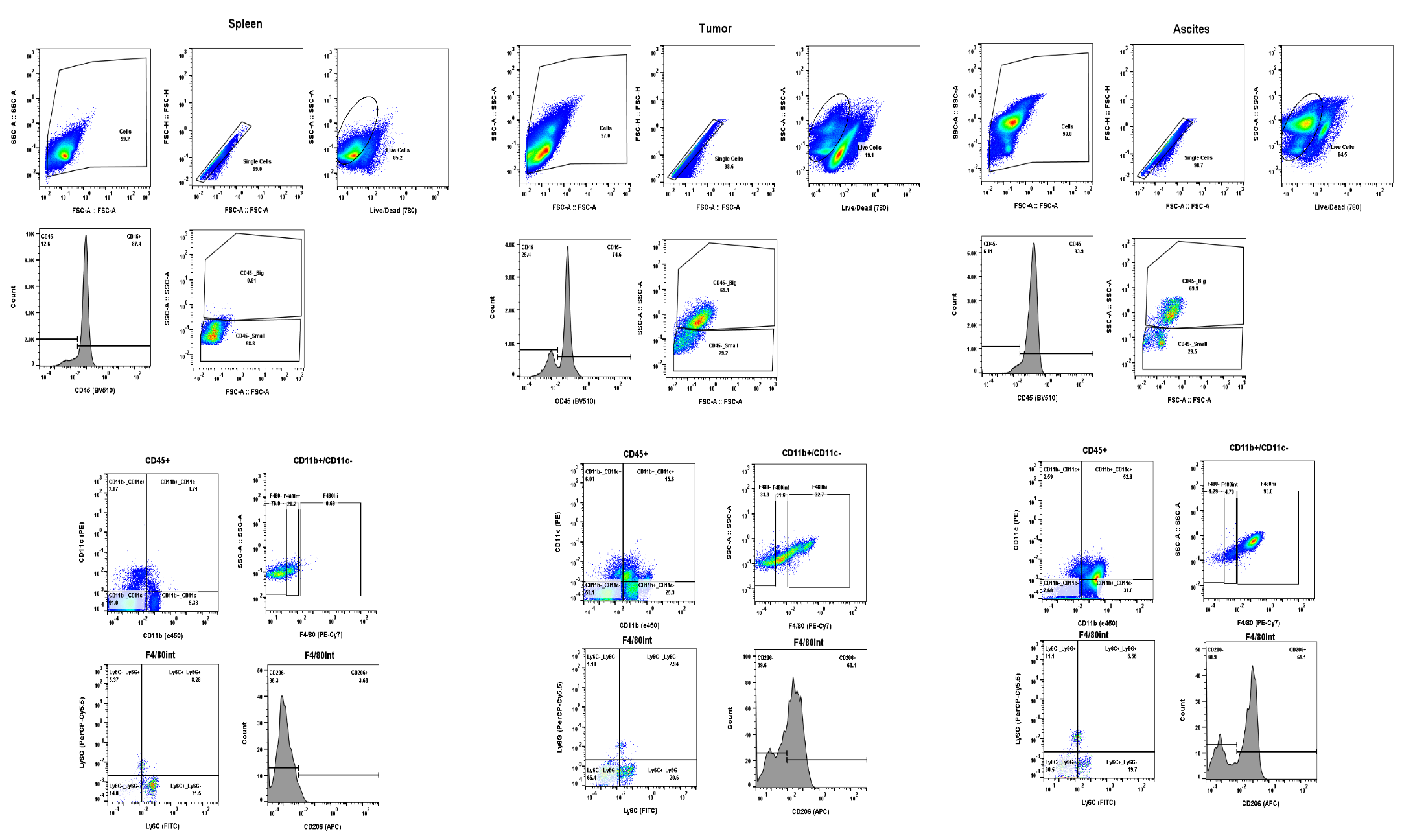


**Solid Tumor**

**Ascites**

**Spleen**

**Supplemental Table 2:** qRT-PCR primer sequences. IκBα, IL-6, CXCL9, CD206, B2M, and GAPDH were purchased from IDT. Arginase-1 and TNF-α were purchased from Sigma.

| **Target Gene** | **Forward Sequence (5’🡪3’)** | **Reverse Sequence (5’🡪3’)** |
| --- | --- | --- |
| **IL-6** | GATCCGGTGATAGTTCCTCTTCA | TCCAGAGTCAGCGCCTCATA |
| **CD206** | CAAGGAAGGTTGGCATTTGT | CCTTTCAGTCCTTTGCAAGC |
| **IκBα** | GAGCTCCGAGACTTTCGAGG | AGACACGTGTGGCCATTGTA |
| **Arginase-1** | CTCCAAGCCAAAGTCCTTAGAG | AGGAGCTGTCATTAGGGACATC |
| **CXCL9** | GTGGTGAAATGGAAAGATCAGGGC | AAGAGAGAAATGGGTTCCCTGGAG |
| **TNF-α** | CCCTCACACTCAGATCATCTTCT | GCTACGACGTGGGCTACAG |
| **CCL3** | TGCCCTTGCTGTTCTTCTCT | GATGAATTGGCGTGGAATCT |
| **B2M** | CTGCTACGTAACACAGTTCCACCC | CATGATGCTTGATCACATGTCTCG |
| **GAPDH** | CCCTTAAGAGGGATGCTGCC | TACGGCCAAATCCGTTCACA |

**Supplemental Figure S12:** Uncropped images of western blot. The blot was cut into three pieces prior to antibody probing to allow for probing of multiple proteins of different sizes on the same blot (the loading control was run on the same blot as experimental antibody). Shown are the three cut pieces of the same blot consisting of (A) the loading area, (B) IκBα (39 kDa), and (C) Histone H3 (15 kDa).

A

B

C


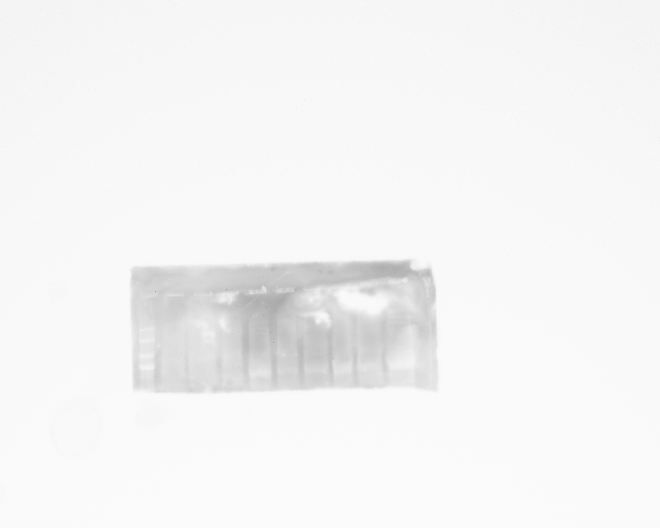

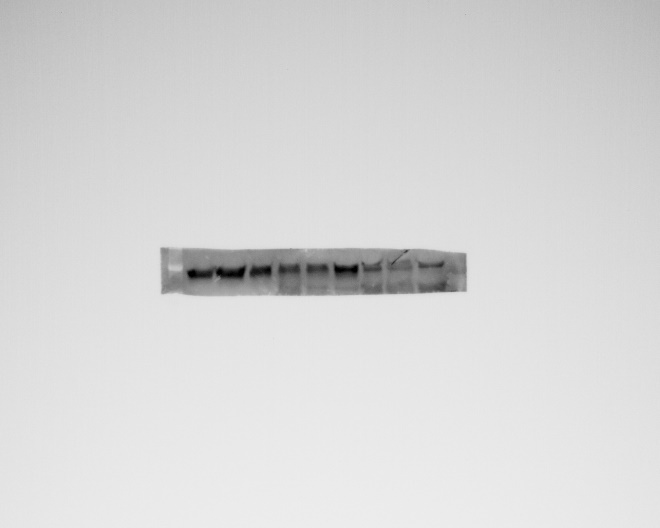

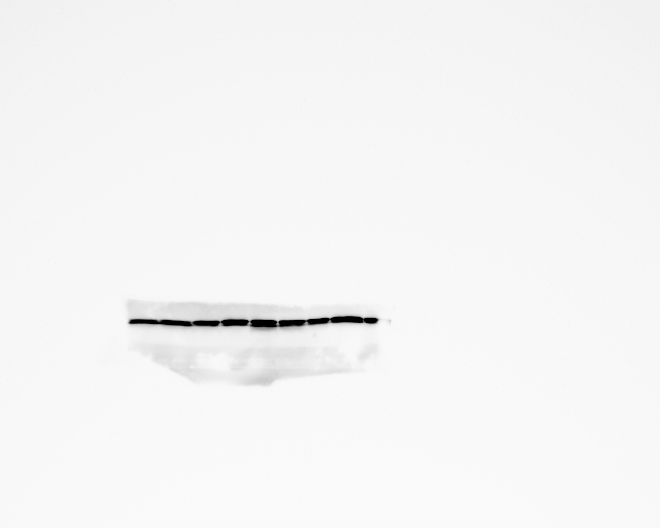

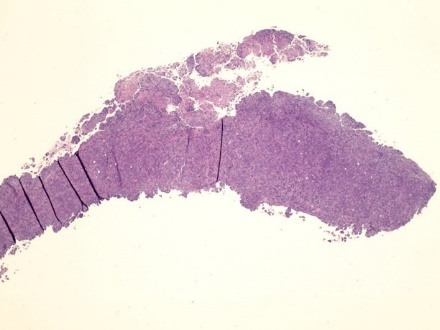

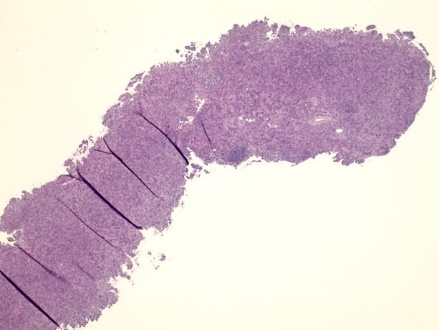

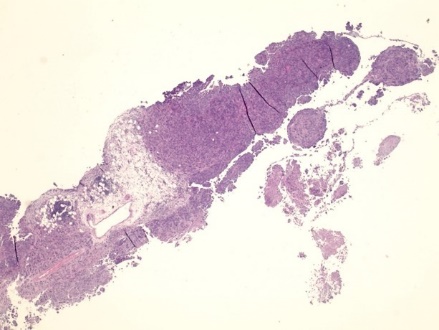


PBS Control Scr-MnNP IκBα-MnNP

**Supplemental Figure S13:** Solid tumors from the ID8 model were sectioned, stained with H&E, and imaged at 4X. The PBS control and Scr-MnNP tissue appeared to be all healthy tumor cells, while decreases in epithelial cellularity was evident in the IκBα-MnNP treatment, possibly indicating a decrease in tumor cells.

**Supplemental Figure S14:** RNA isolated from ascites and tumor cells was used for qRT-PCR analysis. (**A**) CXCL9, a chemokine released by M1 macrophages that attracts Th1 cells, expression in the ascites increased in the IκBα-MnNP treatment. RNA from the tumor revealed slight increases in (**B**) TNF-α and (**C**) CCL3 expression, both inflammatory cytokines.


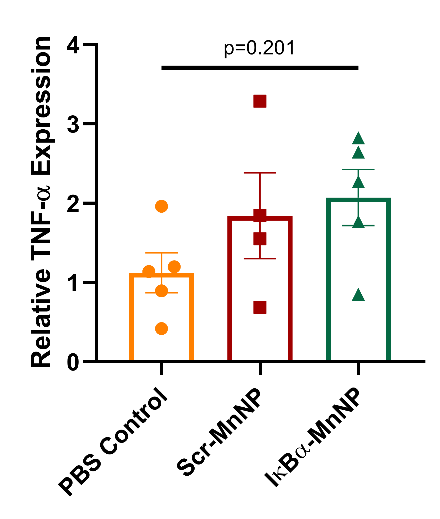

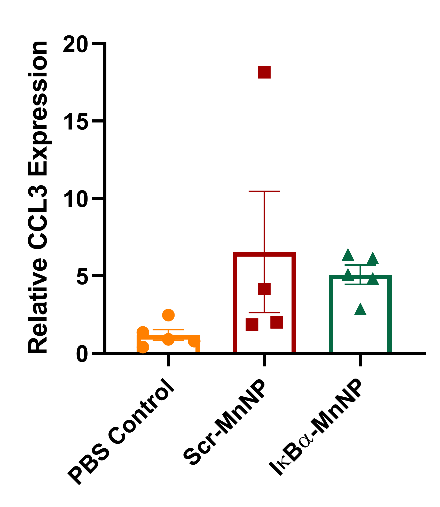

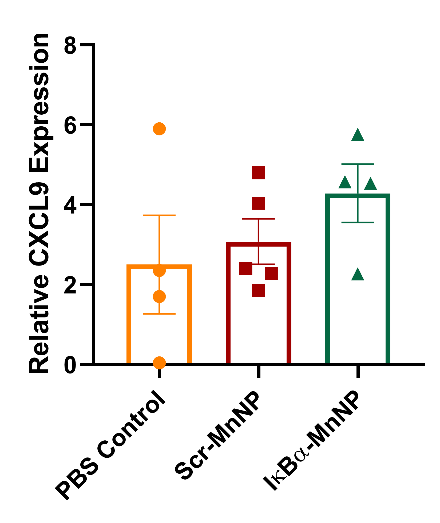


Ascites Tumor

A

B

C


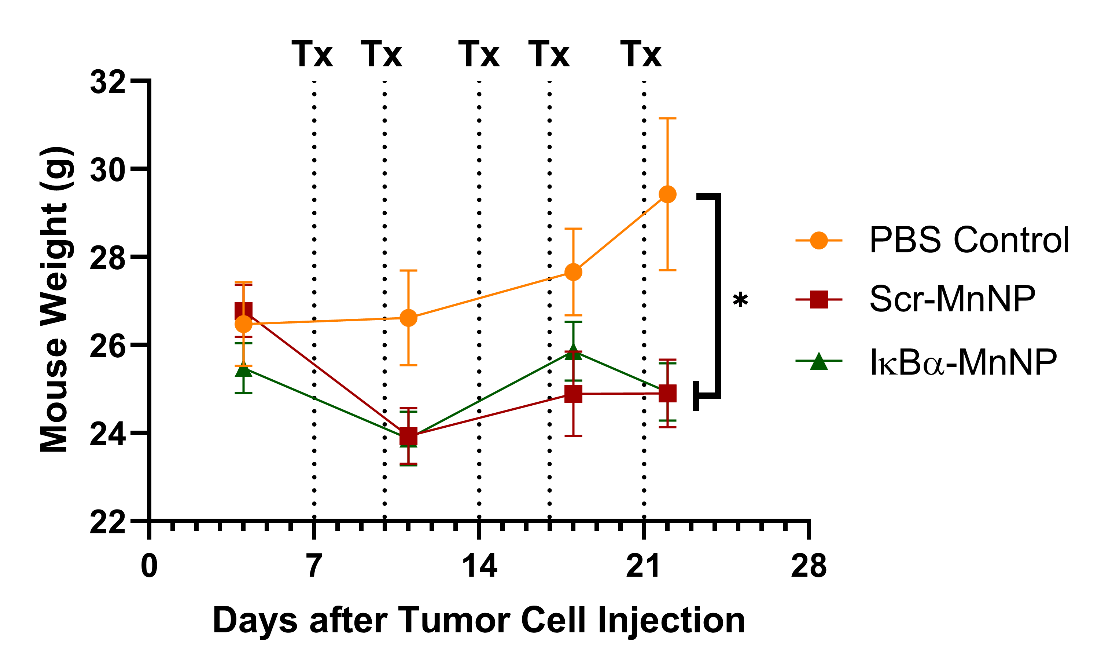


**Supplemental Figure S15:** Change in overall mouse weight over the course of tumor development and MnNP treatment of TBR5-bearing mice. Scr-MnNP and IκBα-MnNP significantly reduced weight gain associated with ascites development compared to PBS control (n=5, *p<0.05).

**Supplemental Figure S16:** Solid tumors from the TBR5 model were sectioned, stained with H&E, and imaged at 4X. The PBS control tissue appeared to be all healthy epithelial cells, while loss of epithelial cellularity was evident in the Scr-MnNP treatment. The IκBα-MnNP treatment appeared to show signs of immune cell infiltration (yellow circles).


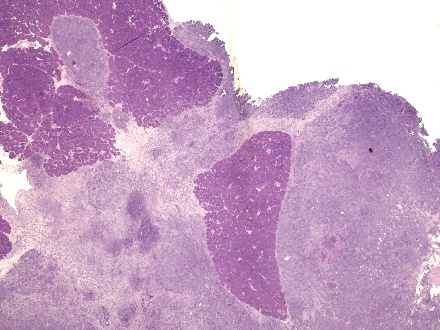

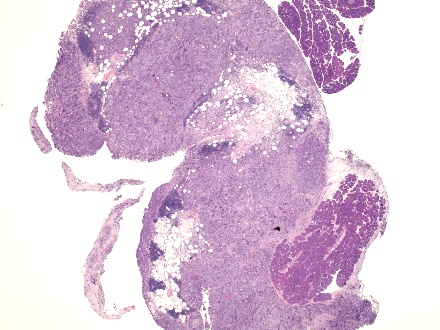

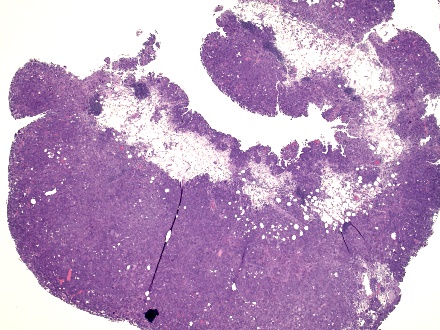


PBS Control Scr-MnNP IκBα-MnNP

**Supplemental Figure S17:** RNA isolated from the ascites and tumor cells was used for qRT-PCR analysis to evaluate expression of IκBα. IκBα expression increased in the ascites samples treated with Scr-MnNP, but this increase was offset in the IκBα-MnNP treatment. No significant changes were observed in the tumors, although there was a trend of decreasing IκBα expression in the IκBα-MnNP treatment.

Ascites Tumor


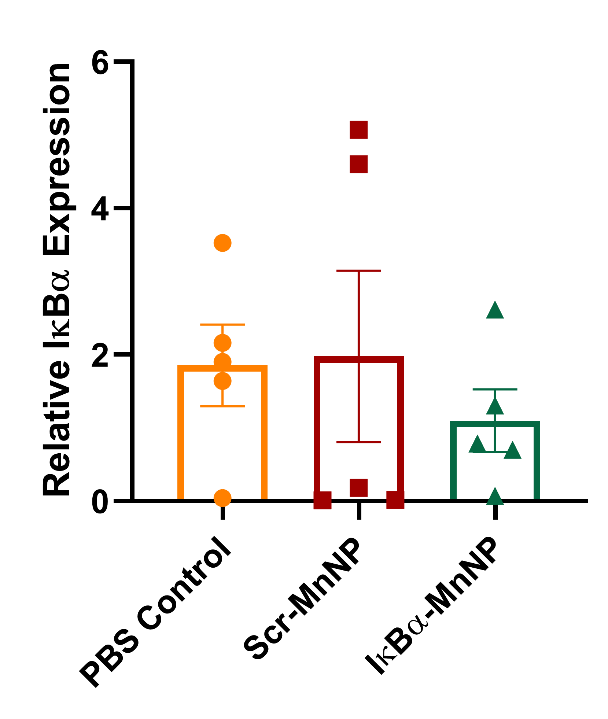

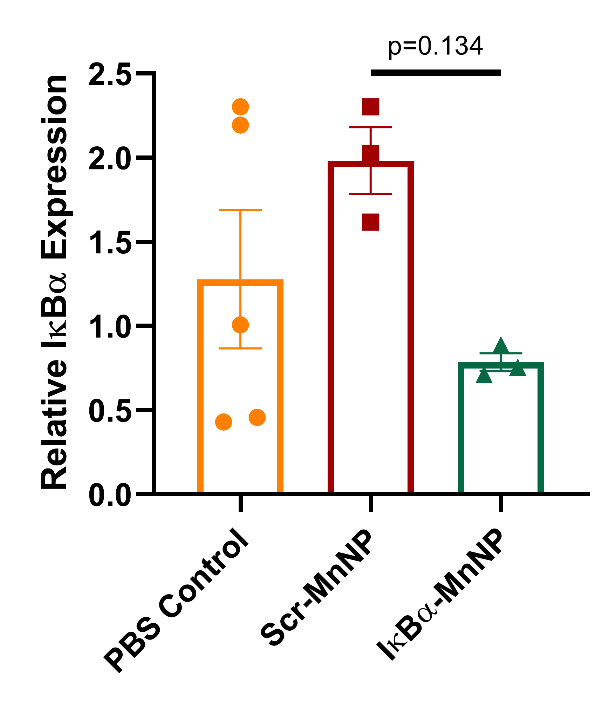

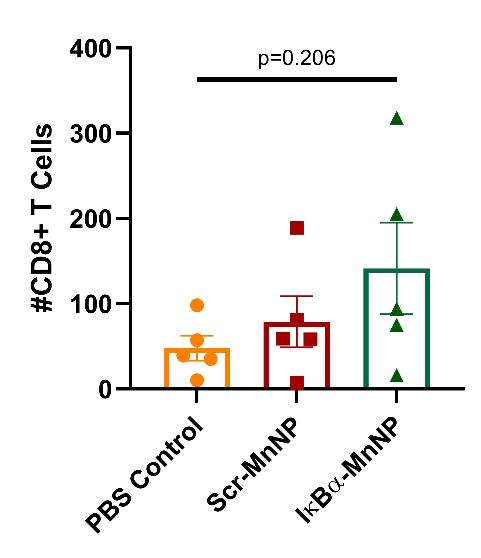

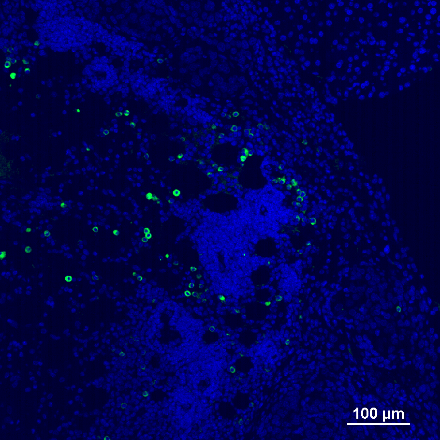

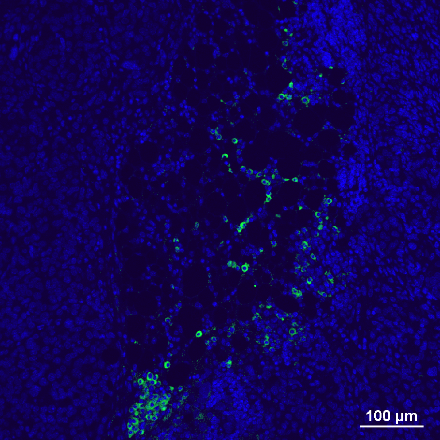

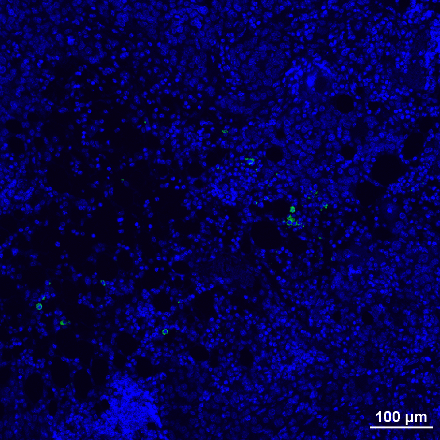


PBS Control Scr-MnNP IκBα-MnNP

**Supplemental Figure S18:** TBR5 tumors were sectioned and stained for cell nuclei (DAPI-blue) and CD8 (Alexa Fluor 488-green). Representative images revealed limited CD8 T cell presence in the control tumors, but both MnNP treatments induced CD8 T cell infiltration. Quantification of CD8+ T cells revealed a trend in increasing T cell infiltration in the IκBα-MnNP treatment compared to the PBS control and Scr-MnNP treatment.


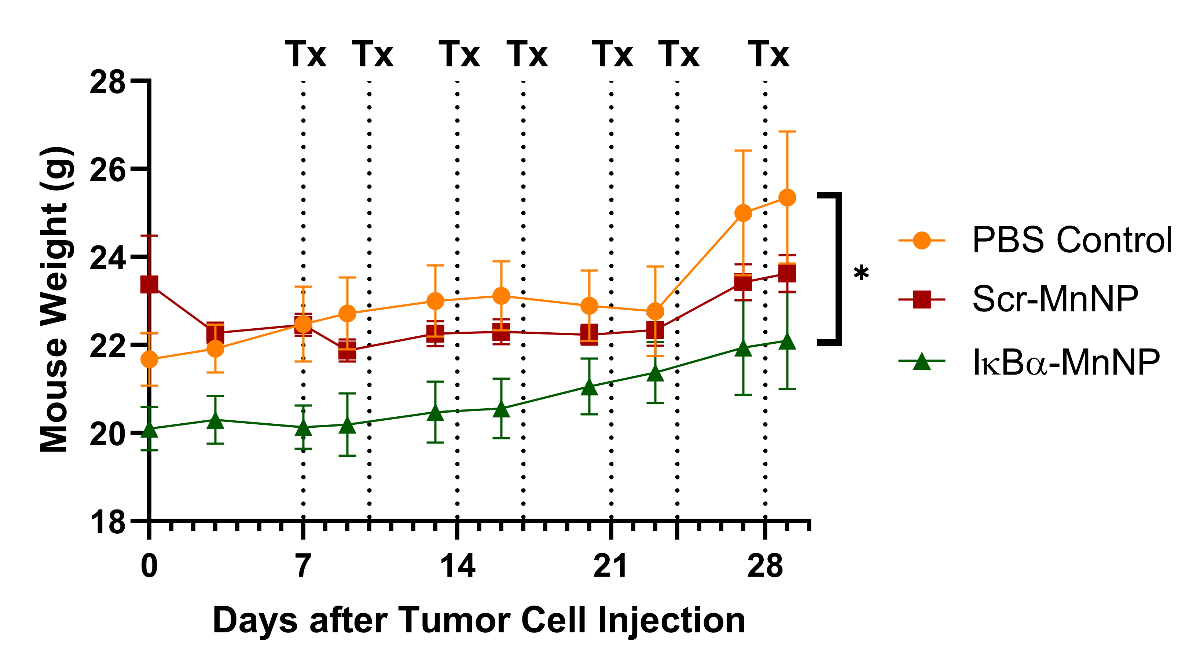


**Supplemental Figure S19:** Mouse weights over the course of the extended biweekly treatment in the TBR5 model revealed a steady increase in the PBS control mice attributed to ascites accumulation which was not observed in the two treatments. These results match with the MnNP treatments preventing ascites accumulation observed when measuring ascites volume at endpoint.

**Supplemental Figure S20:** Quantification of immune cell composition in the ascites and tumors of TBR5 mice in the extended MnNP treatment revealed positive therapeutic trends in several subsets. These trends included an increase in NK cells, CD8+ T cells, dendritic cells, and M1 macrophages (F4/80int/CD206-) as well as a decrease in CD4+ T cells and M2 macrophages (F4/80int/CD206+).


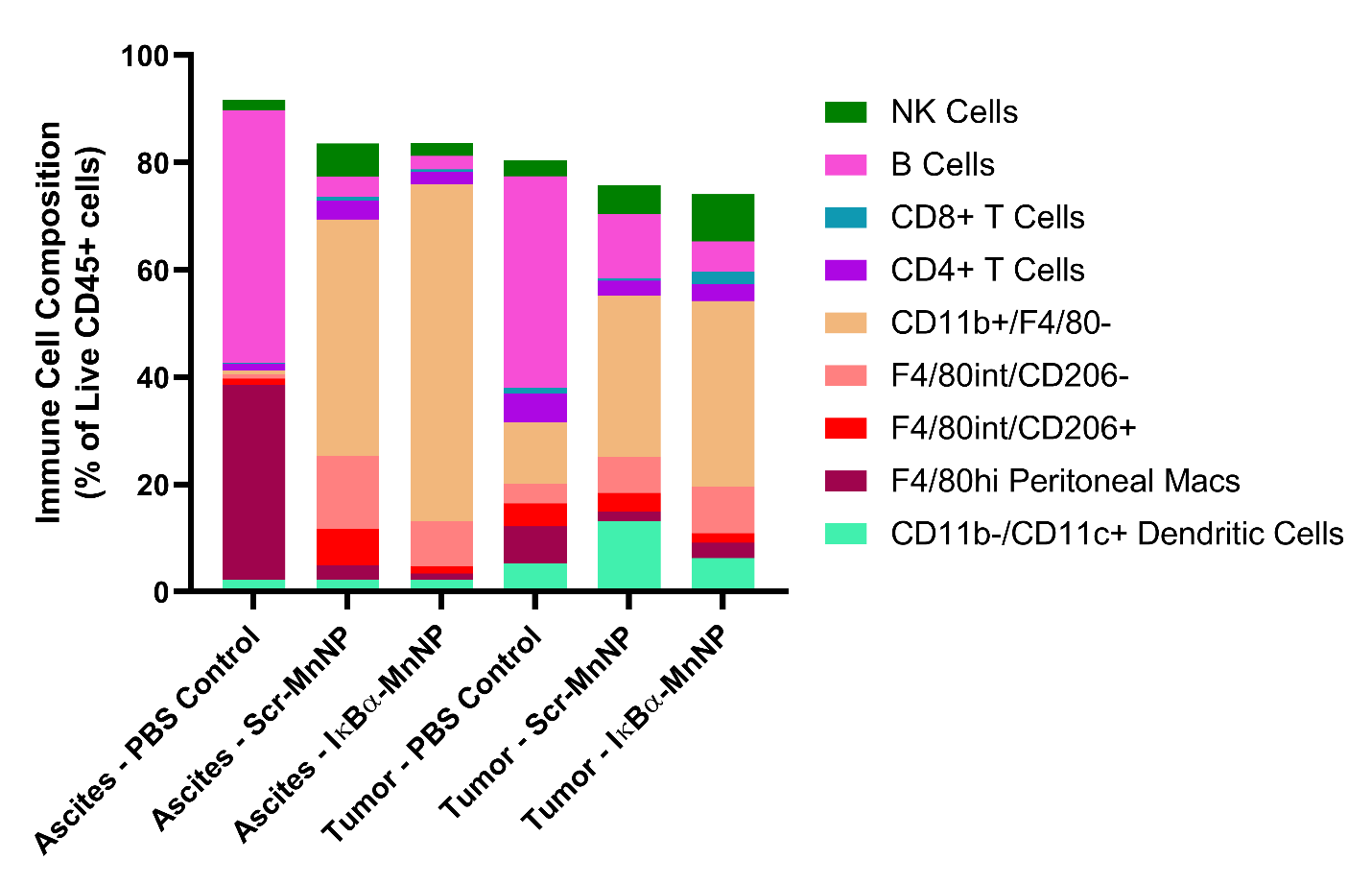

Supplement: Supplementary file 1 — Additional file 1. [file 12885_2022_9612_MOESM1_ESM.docx]
